# The Green Treasure from Appennine Flora for Colon and Liver Health: Characterization and Evaluation of the Protective Effects from Aerial Parts of *Helichrysum italicum*

**DOI:** 10.3390/plants15071108

**Published:** 2026-04-03

**Authors:** Maria Loreta Libero, Gianluca Genovesi, Mariachiara Gabriele, Annalisa Chiavaroli, Giustino Orlando, Luigi Brunetti, Sheila Leone, Lucia Recinella, Gokhan Zengin, Giovanni Caprioli, Laura Acquaticci, Mehmet Veysi Cetiz, Luigi Menghini, Claudio Ferrante, Simonetta Cristina Di Simone

**Affiliations:** 1 Botanical Garden “Giardino dei Semplici”, Department of Pharmacy, “G. d’Annunzio” University “Chieti-Pescara”, Via dei Vestini n. 31, 66100 Chieti, Italy; maria.libero@unich.it (M.L.L.); gianluca.genovese@phd.unich.it (G.G.); mariachiara.gabriele@phd.unich.it (M.G.); annalisa.chiavaroli@unich.it (A.C.); giustino.orlando@unich.it (G.O.); luigi.brunetti@unich.it (L.B.); sheila.leone@unich.it (S.L.); lucia.recinella@unich.it (L.R.); luigi.menghini@unich.it (L.M.); simonetta.disimone@unich.it (S.C.D.S.); 2 Physiology and Biochemistry Research Laboratory, Department of Biology, Science Faculty, Selcuk University, Konya 42130, Turkey; gokhanzengin@selcuk.edu.tr; 3 Chemistry Interdisciplinary Project (CHIP) Research Center, School of Pharmacy, University of Camerino, 62032 Camerino, Italy; giovanni.caprioli@unicam.it (G.C.); laura.acquaticci@unicam.it (L.A.); 4 Department of Medicinal Biochemistry, Faculty of Medicine, Harran University, Sanliurfa 63300, Turkey; mvcetiz@gmail.com

**Keywords:** *Helichrysum italicum*, phenolic compounds, enzyme inhibition, antioxidant, COX-2, IL-6, colon, liver, Docking

## Abstract

*Helichrysum italicum* Mill. (Asteraceae), a perennial evergreen species native to the Mediterranean basin, has been traditionally employed to treat various inflammatory and infectious diseases, as well as respiratory, digestive, gallbladder, and bladder disorders. The plant is valued for its essential oil. It contains phenols and flavonoids, which play a fundamental role in the protective effects associated with the traditional use of extracts of its aerial parts. The goal of the study was to investigate the phytochemical and biological properties of polar extracts, specifically water and hydroalcoholic (50% ethanol) extracts, obtained from the aerial parts of *H. italicum*. The extracts were evaluated for phenolic composition and concurrently assessed for antioxidant and enzyme-inhibitory activities. Additionally, the biocompatibility of the extracts was investigated using eco-toxicological models, including *Artemia salina* lethality and *Daphnia magna* cardiotoxicity assays, as well as allelopathic studies. CCD841CoN colon epithelial cell viability was also assessed in the presence of the extracts. The extracts’ protective effects were examined in an ex vivo inflammatory model using isolated mouse colon and liver tissues exposed to *Escherichia coli* lipopolysaccharide (LPS). Their influence on cyclooxygenase-2 (COX-2) and interleukin-6 (IL-6) gene expression was investigated, as well. Docking studies were also performed to uncover on the potential mechanisms underpinning the biological effects observed in the study. The phytochemical analysis showed that hydroxycinnamic acids and quercetin derivatives were the primary components in both extracts. In particular, the hydroalcoholic extract showed higher phenol levels and more potent scavenging/reducing and enzyme inhibitory activities against tyrosinase, cholinesterases, glucosidase, and amylase. Using the aforementioned eco-toxicological and in vitro cell models, the extracts’ biocompatibility was determined to be in the range of 200–1000 µg/mL. Within this concentration interval, the extracts effectively mitigated LPS-induced stimulation of COX-2 and IL-6 gene expression. Docking studies suggest that hydroxycinnamic acids (notably chlorogenic acid) and flavonoids (including quercetin, rutin, hyperoside, and isoquercitrin) play a pivotal role in the extracts’ anti-inflammatory activity. In conclusion, this study provides scientific evidence supporting the ethnopharmacological use of *H. italicum* in managing oxidative stress and inflammatory disorders, especially in the digestive system. Phenolics in the extracts likely enhance their therapeutic potential. These findings warrant further research, including in vivo studies, to assess the extracts’ efficacy and safety profile comprehensively.

## 1. Introduction

*Helichrysum italicum* (Roth) G.Don, 1830, known as a perennial evergreen species of the *Helichrysum* genus Mill. (Asteraceae), is native to the Mediterranean area [1,2]. More than 1000 taxa are currently recognized within the genus, whose name is derived from the Greek terms helios and chrysos, in reference to the characteristic bright-yellow inflorescences. These inflorescences constitute the principal plant material employed in ethnopharmacological applications [2]. This plant grows as a tall shrub or subshrub and has traditionally been used to treat various inflammatory and infectious conditions, including respiratory, digestive, and biliary disorders, as well as issues affecting the gallbladder and bladder. The first scientific studies were carried out in the first half of XX century, in patients suffering from skin disorders, including psoriasis, whilst more recent studies indicated neuroprotective and antidiabetic effects induced by plant extracts [3,4]. Besides its essential oil content, which comprises neryl acetate and α-pinene as its main components, the plant also contains phenolic and flavonoid compounds, which likely contribute to its protective effects associated with the traditional use [2]. There is increasing interest in the use and properties of medicinal plants. Research is exploring novel pharmacological applications and driving the development of innovative phytotherapy and herbal-based food supplements. *Helichrysum italicum* is also of great interest, with nearly half of the articles exploring the bio-pharmacological uses of this species published and indexed in PubMed in the last five years. This is also due, albeit partially, to the growing concerns over the side effects of synthetic medications [5]. As a result, herbal preparations have gained popularity, particularly for managing mild chronic conditions, thanks to their favourable safety profile. This trend has increased demand for plant-based products, prompting scientific research to establish a rational basis for their use, whether they are already in use as treatments or represent promising discoveries [6]. Additionally, the valorisation of traditional herbal preparations, especially those obtained via infusion and/or decoction, could be a smart and successful strategy for implementing local botanical chains, and promoting the knowledge of biodiversity and agrobiodiversity among the population. In this scenario, the present study evaluated the potential of water and hydroalcoholic (water/ethanol 50/50 *v*/*v*) extracts from the aerial parts of *H. italicum.* The extracts were prepared using laboratory methods that mimic the traditional infusion and maceration procedures used to prepare tea and liqueurs, respectively. This is also consistent with the traditional therapeutic uses of *H. italicum* for respiratory, digestive, and inflammatory conditions, in which infusions and decoctions are most commonly employed [3]. Intriguingly, although polar extracts are the most used for traditional medicine purposes, scientific studies focused on the use of crude extracts are still limited [3]. Therefore, our study pointed to improve scientific evidence about the use of *H. italicum* crude extracts in modern phytotherapy. The water and hydroalcoholic extracts were analyzed to determine their phenolic composition and evaluate their antioxidant and enzyme-inhibiting properties. The extracts’ biocompatibility was assessed using ecotoxicological models, including allelopathic assays, *Artemia salina* (brine shrimp) lethality and *Daphnia magna* cardiotoxicity assays. Additionally, the effects of the extracts on non-tumoral CCD841CoN colon epithelial cell viability were determined. The protective effects of the extracts were then investigated in an ex vivo inflammatory model, in which isolated mouse colon and liver tissues were exposed to *Escherichia coli* lipopolysaccharide (LPS). The extracts’ modulation of COX-2 and IL-6 gene expression was subsequently evaluated. To further elucidate the molecular mechanisms, docking studies were undertaken.

## 2. Results and Discussion

### 2.1. Phytochemical Analysis

In this work, water and hydroalcoholic (50/50% *v*/*v*) extracts from *H. italicum* were investigated for their composition and biological activity. The two extracts were chosen based on the traditional use of the plant. Indeed, the aerial parts are usually employed in the preparation of infusions/decoctions [2] and liqueurs, particularly in Sardinia (ITALY), to ameliorate gastrointestinal function. Phenolic compounds exhibit remarkable biological properties and are being studied for their potential health benefits. Total flavonoids and phenolics from *H. italicum* extracts were quantified via colorimetric methods (Table 1). The ethanol (50%) extract showed the highest concentrations of total flavonoids and phenols, with values of 62.05 mg RE/g and 86.59 mg GAE/g, respectively. The findings revealed that flavonoids constituted approximately 80% of the total phenolic compounds. Additionally, the hydroalcoholic solution proved to be more effective, likely due to its lower polarity and capacity to facilitate the dissolution of phenolic compounds. Previous reports on the genus *Helichrysum* showed the potential use of a hydroalcoholic mixture for health-promoting applications [7]. Various studies suggested different levels of total phenols and flavonoids in *H. italicum*. For instance, Nebrigic et al. (2023) found total phenols ranging from 49.70 to 119.77 mg GAE/g and flavonoids ranging from 2.92 to 21.96 mg RE/g [8]. CuriĆ et al. (2025) reported total phenolic content of 64.5–75.1 mg GAE/g and flavonoid content of 17.4–20.4 mg CAE/g [9]. Similarly, studies on *H. arenarium* showed varying levels of phenolic compounds, with leaf extracts containing more than flower extracts [10]. Methanol extracts of *H. armenium* were also found to be rich in phenolic and flavonoid content [11]. These variations can be due to geographical and climatic conditions, as well as plant parts and standards employed for interpretation. Additionally, concerns have been raised about the quantification of total phenolic compounds by colorimetric methods, and the results need confirmation by chromatographic methods. Not only phenolic compounds, but also peptides, can reduce the Folin–Ciocalteu reagent, leading to inaccurate quantification [12]. In this scenario, the extracts’ phytochemicals were quantified using HPLC-ESI-MS/MS. The chromatographic analysis of 38 compounds belonging to various phenol classes (phenolic acids, hydroxycinnamic acids, anthocyanins, and flavonoids) showed similar results. The chromatographic analysis indeed confirmed that the hydroalcoholic extract was the richest in phenolic compounds. Particularly abundant were hydroxycinnamic acids, such as chlorogenic acid, and flavonoids, such as quercetin and its glycosylation products, namely rutin, hyperoside and isoquercitrin (Table 2). Our findings agree, at least in part, with the results reported by Bojilov et al. (2023) that identified quercetin and chlorogenic acid derivatives as the main phenolic compounds in extracts from aerial parts of *H. italicum* [13]. Intriguingly, this is also consistent, albeit in part, with the phytochemical results obtained by Buyukyildirim and colleagues (2025) [14]. Indeed, they found chlorogenic acid and isoquercitrin among the prominent phytochemicals present in the aerial parts’ extracts from another species of the *Helichrysum* genus, namely *H. plicatum* [14].

### 2.2. Antioxidant Effects

Antioxidant compounds are currently among the most fascinating molecules, with applications ranging from pharmaceuticals to nutraceuticals. One of the main reasons for this situation is that they neutralize free radicals and improve the prognosis of many diseases [15]. In this context, the discovery of new, effective, and natural antioxidant sources is highly valuable for providing raw materials for these applications. In the present research, the scavenging/reducing properties of *H. italicum* extracts were investigated through ABTS, DPPH, FRAP, CUPRAC, phosphomolybdenum, and chelating assays. The results are depicted in Table 3. In ABTS and DPPH assay, the highest radical scavenging capacities were observed in the hydroalcoholic extract, with 271.44 mg TE/g and 310.52 mg TE/g, respectively. A similar trend was observed for the CUPRAC and FRAP assays, with the highest values in the hydroalcoholic extract, reflecting its electron-donating ability. Similarly, the most active extract for chelation of transition metals was the hydroalcoholic extract, with 16.86 mg EDTAE/g. Overall, the antioxidant results mirror the trend of total phenolic and flavonoid levels in the extracts. This implies that phenols play a key role in the antioxidant activity of the extracts. In addition, Table 2 shows that the levels of some compounds, including rutin [16], hyperoside [17], quercetin [18], and chlorogenic acid [19], in the hydroalcoholic extract were higher than those in the water extracts, which may explain why this extract was the most active. A previous research has reported antioxidant properties of species in the genus *Helichrysum*, including *H. italicum.* In agreement with our findings, Kramberger et al. (2020) reported that the hydroalcoholic extracts of *H. italicum* exhibited the strongest antioxidant activity [20]. In another study, Gevrenova et al. (2023) investigated the antioxidant properties of a hydromethanolic extract from *H. italicum* and that extract (DPPH: 110.33 mg TE/g; ABTS: 234.70 mg TE/g; CUPRAC: 354.23 mg TE/g; and FRAP: 210.4 mg TE/g) demonstrated lower activity than the extract evaluated in our study [3]. Talić and colleagues examined the antioxidant properties of three parts of *H. italicum* (whole plants, flowers, and leaves) using DPPH and FRAP assays [21]. They found that flower extracts were the most active, with the lowest IC_50_ value of 23 µg/mL. In another study by Nebrigic et al. (2023), the DPPH and ABTS radical abilities of the tested extracts ranged from 38.70 to 149.49 mg TE/g and from 114.51 to 239.34 mg TE/g, respectively [8].

### 2.3. Enzyme Inhibition Effects

In recent times, the global burden of health problems has been constantly increasing. For example, the prevalence of diabetes has almost doubled from 2015 to 2025. In this sense, the effective therapeutic strategy needs to manage these diseases. In the strategy, enzymes are the main players, and inhibiting key enzymes can alleviate symptoms. For instance, most oral antidiabetics contain α-amylase and α-glucosidase inhibitors, which retard the postprandial rise in glucose in patients with diabetes [22]. Additionally, cholinesterase inhibitors are first-line treatments for Alzheimer’s disease and aim to increase acetylcholine levels at the synaptic cleft, thereby enhancing memory function [23]. Given the importance of enzyme inhibitors, several compounds have been used as such. While these inhibitors are effective, they are often associated with significant side effects, including toxicity, gastrointestinal distress, and an increased risk of long-term resistance or reduced efficiency. Thus, alternative sources of enzyme inhibitors are gaining interest in the pharmaceutical field. The enzyme-inhibitory effects of *H. italicum* extracts were assessed using in vitro assays, and the results are described in Table 4. The highest AChE inhibition was found in the hydroalcoholic extract with 2.18 mg GALAE/g. However, the best BChE inhibition was observed in the water extract with 1.10 mg GALAE/g. Once again, the hydroalcoholic extract provided the best tyrosinase inhibition.

Regarding antidiabetic enzymes, the highest value was found in the hydroalcoholic extract. The chemical profile of the hydroalcoholic extract explains its remarkable enzyme-inhibitory effect. In particular, the presence of some components in the hydroalcoholic extract can contribute to its ability as an enzyme inhibitor. For example, rutin is known as an effective inhibitor of AChE [24], tyrosinase [25], and α-amylase [26]. In addition, hyperoside and quercetin exhibited significant enzyme inhibitory effects, as described by previous studies [27,28,29]. Previous studies have also reported enzyme inhibitory effects of *Helichrysum* species, including *H. italicum*. For example, Gevrenova et al. (2023) showed AChE and BChE inhibitory effects of 1.64 mg GALAE/g and 0.11 mg GALAE/g, respectively, which were lower than those described in the present study [3]. Talić et al. (2019) showed that *H. italicum* flower extract had the strongest AChE inhibitory effect compared to leaves and whole plant [21]. Ethyl acetate and methanol extracts of *H. armenianum* displayed significant tyrosinase inhibition [11]. Overall, these results indicate that *H. italicum* could represent a valuable source of enzyme inhibitors for therapeutic applications.

### 2.4. Biocompatibility Evaluation

#### 2.4.1. Eco-Toxicological Assays

**Figure 1:**
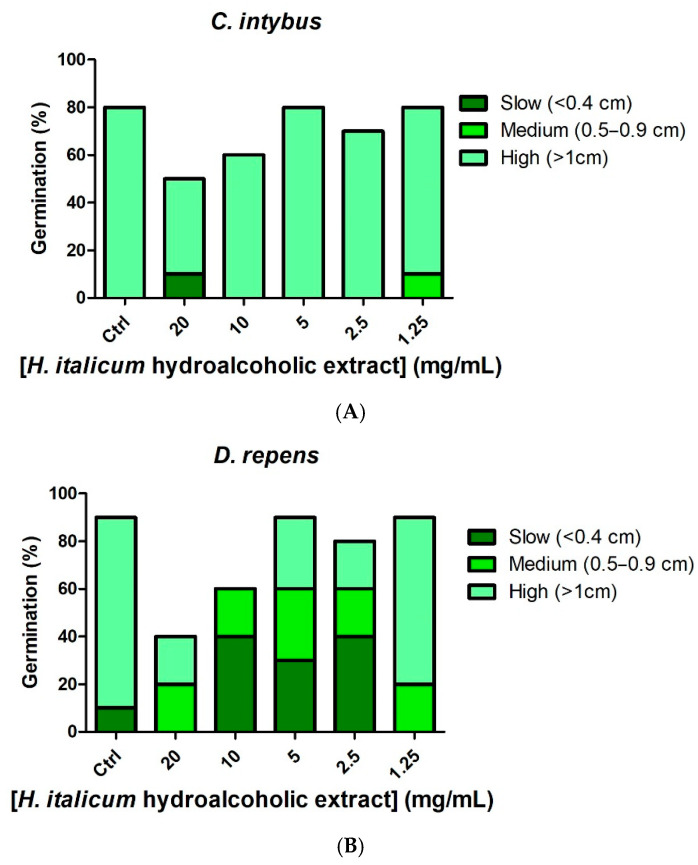

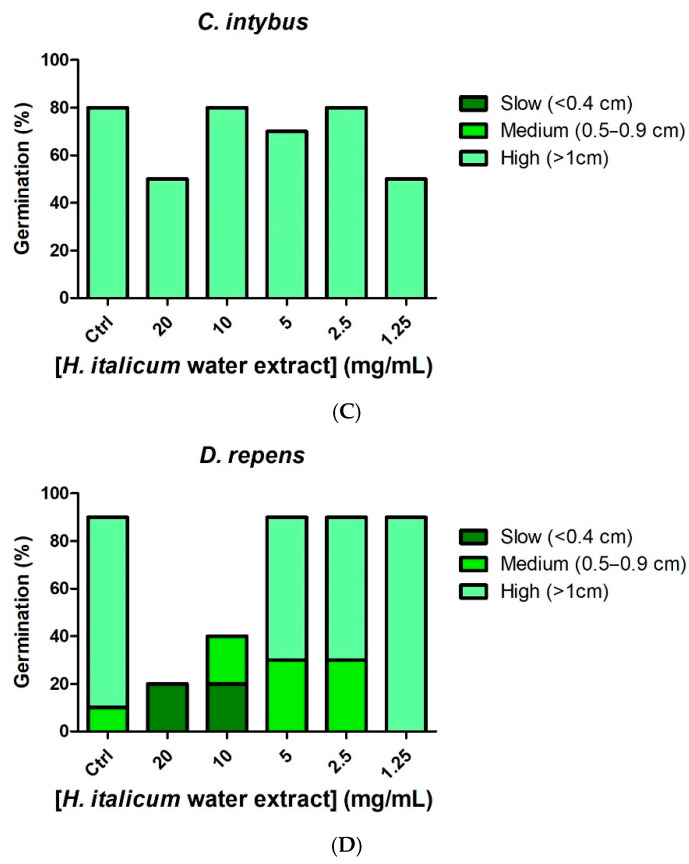
(**A**): Effect of *Helichrysum italicum* hydroalcoholic extract (1.25–20 mg/mL) on *Cichorium intybus* seedling germination. (**B**): Effect of *Helichrysum italicum* hydroalcoholic extract (1.25–20 mg/mL) on *Dicondra repens* seedling germination. (**C**): Effect of *Helichrysum italicum* water extract (1.25–20 mg/mL) on *Cichorium intybus* seedling germination. (**D**): Effect of *Helichrysum italicum* extract (1.25–20 mg/mL) on *Dicondra repens* seedling germination.

Allelopathy refers to the biological phenomenon whereby plants release phytochemicals, or allelochemicals, that influence the growth and development of nearby plants. These chemicals are present in various parts of plants and released through processes such as volatilization and root exudation [30]. Understanding allelopathy sheds light on the interactions between plants and ecosystems. The allelopathic potential of water and hydroalcoholic extracts of *H. italicum* was assessed by examining their impact on seed germination and growth of *Dichondra repens* and *Cichorium intybus*. The results showed concentration-dependent inhibition of seed growth in both species, regardless of the type of extract used. In the control groups, high germination percentage values were observed for both *C. intybus* and *D. repens*, with most seeds exhibiting high growth rates (>1 cm). This established a baseline for evaluating the extracts’ inhibitory effects. At higher concentrations (20 mg/mL) of both hydroalcoholic and water extracts, a significant decrease in germination percentage was observed. For instance, the hydroalcoholic extract reduced the germination percentage of *C. intybus* to around 50% (Figure 1A). The germination percentage of *D. repens* was approximately 40% (Figure 1B). Similarly, the water extract at the same concentration resulted in a germination percentage reduction to about 50% for *C. intybus* (Figure 1C) and 20% for *D. repens* (Figure 1D). These reductions indicate a pronounced allelopathic effect. At a concentration of 10 mg/mL, the inhibitory effects were still evident but less pronounced than at 20 mg/mL. The germination percentage for both species increased slightly, yet remained significantly lower than the control, except for the water extract against *C. intybus*, which did not exhibit a significant inhibitory effect. Lower concentrations (5, 2.5, and 1.25 mg/mL) of both extracts had no effect on germination percentage, with values comparable to the control groups, and most seeds achieved high growth rates. The consistent results observed across both extract types and plant species highlight the allelopathic potential of *H. italicum* at high concentrations. This finding suggests that specific allelochemicals responsible for the inhibitory effects are present in substantial amounts in both types of extracts. These findings also align with the composition of the extracts, which are rich in phenolic acids and hydroxycinnamic acids, both known for their allelopathic properties [31]. Additionally, another *Helichrysum* species, namely *H. arenarium*, displayed significant allelopathic effects, with seed germination inhibitory effects occurring even at lower concentration, 5 mg/mL, compared with the present extracts. This was related, albeit partially, to the presence of flavonoid compounds, especially naringenin, the aglycon form of naringin [32]. We cannot exclude that the absence of this compound in our extracts could scale back the phytotoxic properties of *H. italicum* extracts, compared with *H. arenarium.*

**Figure 2:**
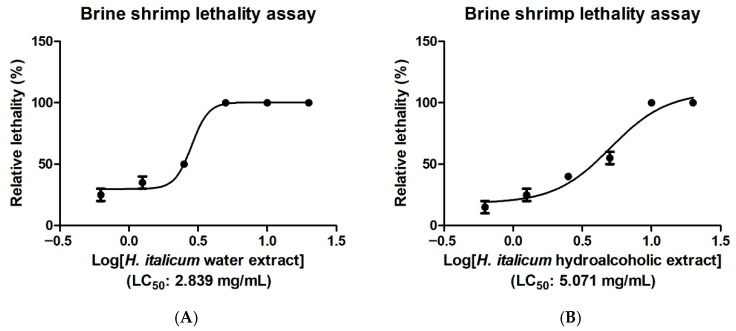
Dose–response curve displaying the lethality effects induced by *Helichrysum italicum* water (subfigure (**A**)) and hydroalcoholic (subfigure (**B**)) (0.625–20 mg/mL) on brine shrimps (*Artemia salina*). LC_50_ values were 2.839 mg/mL and 5.071 mg/mL, respectively.

**Figure 3:**
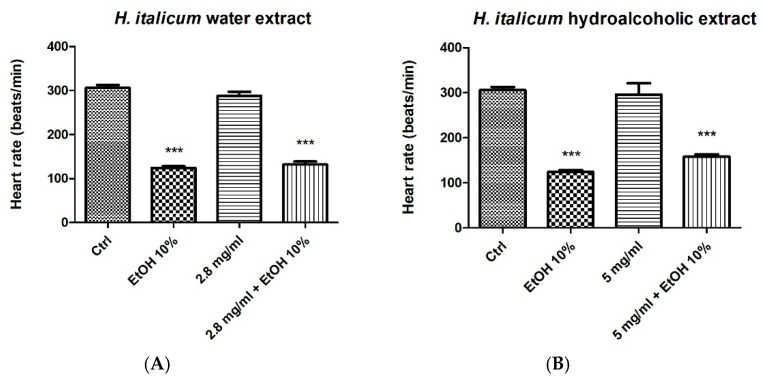
Acute exposure of *Daphnia magna* to *Helichrysum italicum* water (**A**) and hydroalcoholic (**B**) extracts at 2.839 mg/mL and 5.071 mg/mL, respectively, showed no cardiotoxic effects. Co-treatment with 10% ethanol (cardiotoxic stimulus) did not reveal any cardioprotective effects. Data are means ± SEM. ANOVA, *p* < 0.0001. *** *p* < 0.001 vs. respective control group.

The brine shrimp *Artemia salina* is a crustacean inhabiting saline environments and has long been employed in ecotoxicological studies [33,34]. It represents an alternative animal model [35,36] that enables preliminary toxicological screening of a broad spectrum of chemicals, including heavy metals, pesticides, nanoparticles, and natural products, among which herbal extracts [37,38,39]. The potential toxicity of xenobiotics is determined in the brine shrimp using Meyer and Clarkson’s classifications. Meyer et al. (1982) classify non-toxic and toxic substances based on LC_50_ values above and below 1000 µg/mL, respectively [34]. Clarkson et al. (2004) propose to differentiate diverse grades of toxicity as follows: non-toxic; LC_50_ > 1000 µg/mL; low toxic: 500 µg/mL < LC_50_ < 1000 µg/mL; moderate toxic: 100 µg/mL < LC_50_ < 500 µg/mL; highly toxic: LC_50_ < 100 µg/mL [40]. Results (Table 5; Figure 2) show that both water and hydroalcoholic extracts can be classified as biocompatible (non-toxic) against nauplii, with LC_50_ values of 2.84 mg/mL and 5.07 mg/mL, respectively, whilst a previous study demonstrated the high toxicity of the essential oils from both *H. italicum* and *H. arenarium* in the same toxicological model with LC_50_ values in the range 16–24 µg/mL [10]. These results were also consistent with the biocompatibility limits (1.25–5 mg/mL) displayed by the extracts in the allelopathy assay. At concentrations corresponding to the respective LC_50_ values in the *A. salina* lethality assay, *H. italicum* extracts were also tested in the *Daphnia magna* cardiotoxicity assay. In this experimental model, *D. magna* was exposed to the extracts under basal conditions or in the presence of 10% ethanol, which was used to induce a cardiotoxic stimulus. Both extracts did not alter basal heart beat rate, in the crustacean, although they were not effective in blunting the ethanol-induced decrease in hearth beat rate (Figure 3). Intriguingly, the essential oil from *H. italicum* also displayed low to no risk in the acute toxicity assay in the *D. magna* model, with no toxic effects up to concentrations of 800 µg/mL [41]. This concentration value is much higher compared to the LC_50_ value shown by the essential oil in the *A. salina* model [10]. The result may depend on differences in the composition of the essential oil, for example those arising from environmental factors [41]. It may also be influenced by the lower sensitivity of *D. magna* to xenobiotic-induced acute toxicity compared with *A. salina* [42].

#### 2.4.2. CCD841CoN Cell Viability

**Figure 4:**
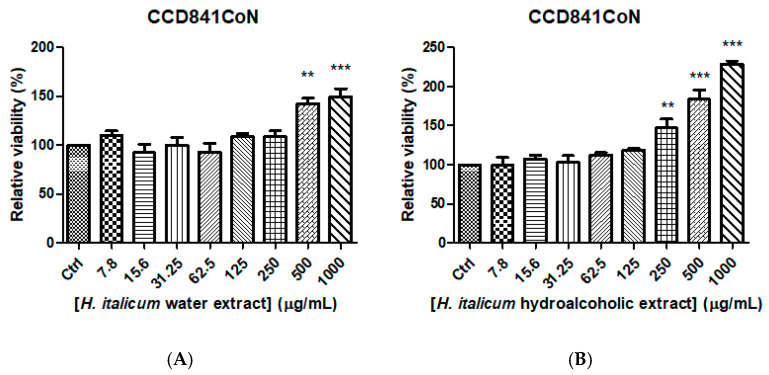
Effects of water (subfigure (**A**)) and hydroalcoholic (subfigure (**B**)) extracts (7.8 µg/mL) from *Helichrysum italicum* on non-tumoral colon CCD841CoN cell viability. ANOVA, *p* < 0.0001; ** *p* < 0.01; *** *p* < 0.001 vs. respective Ctrl (Control) group.

Based on the results of the ecotoxicological assessments, a concentration range ≤ 1000 μg/mL was selected for the subsequent biocompatibility evaluation. In particular, the effects of the extracts on the viability and metabolic activity of the non-tumoral CCD841CoN cell line, utilized as a model of normal gastrointestinal epithelial cells, were evaluated. In the MTT assay, water and hydroalcoholic extracts (7.8–1000 µg/mL) were not cytotoxic. This is consistent with the biocompatibility shown by *H. italicum* infusion in colon fibroblasts [43]. Additionally, the extracts stimulated cell viability in a concentration-dependent manner, especially at the highest tested concentration (Figure 4). This could be possibly related to a putative stimulatory effect on mitochondrial metabolism [44]. Therefore, the present viability assay further corroborates the biocompatibility of the extracts and substantiates their suitability for subsequent evaluation of pharmacological activity in isolated tissues.

### 2.5. Anti-Inflammatory Effects in Isolated Colon and Liver Tissues

**Figure 5:**
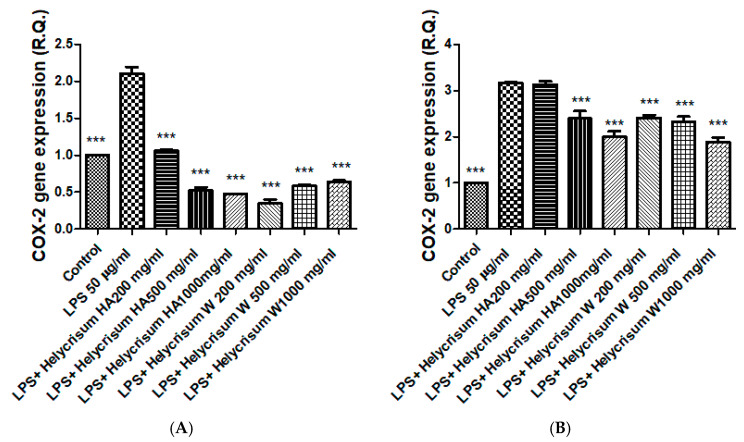

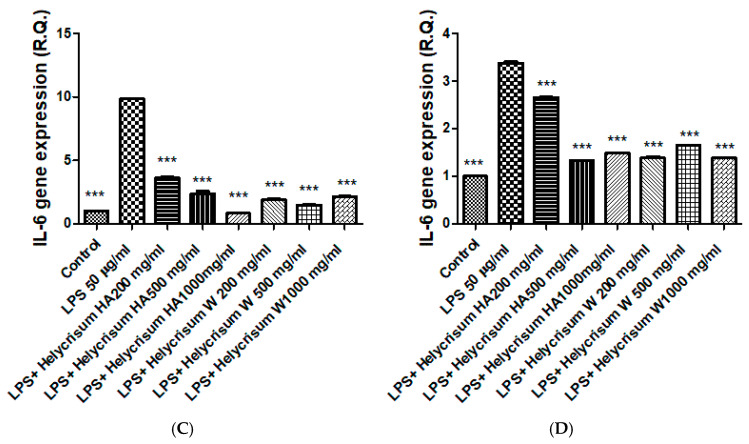
Water and hydroalcoholic extracts of *Helichysum italicum* reduced COX-2 gene expression in LPS-exposed colon (**A**) and liver (**B**) tissues. They also reduced IL-6 gene expression in LPS-exposed colon (**C**) and liver (**D**) tissues. ANOVA *p* < 0.0001; *** *p* < 0.001 vs. respective LPS group.

Protective effects of the extracts (200–1000 µg/mL) were studied on mouse isolated colon and liver tissues. In this context, the goal was to examine the extracts’ ability to reduce inflammation in isolated colon and liver samples exposed to *E. coli* LPS, a validated ex vivo model for evaluating the anti-inflammatory effects of phytocomplexes [45]. Both extracts were effective in reducing LPS-induced upregulation of COX-2 and IL-6 gene expression (Figure 5A–D). These results are consistent with our findings of antioxidant effects displayed by both extracts and their phenolic compound content [46]. Our findings also align with the COX-2 inhibition induced by the ethanolic from *H. odoratissimum,* in murine macrophages and hepatocytes [47]. In this context, hydroxycinnamic acids (e.g., chlorogenic acid) and flavonoids (e.g., quercetin and rutin) could play a key role. These compounds are known to inhibit COX-2 and IL-6 expression [48,49,50]. However, we cannot exclude the possibility that other phytochemicals may contribute, at least in part, to the antioxidant and anti-inflammatory capabilities of the *H. italicum* extracts studied here. Indeed, various acetophenone derivatives isolated from the aerial parts of the plant have demonstrated efficacy in reducing intestinal motility and inflammation in mice [51,52]. In addition, these compounds were shown to inhibit lipid peroxidation in rat liver microsomes exposed to Fe^2+^/ascorbate [53]. These findings collectively provide further support for the traditional use of homemade *H. italicum* preparations in treating and alleviating gastrointestinal and liver disorders [14].

### 2.6. In Silico Results

The in silico analyses presented here are intended to provide mechanistic support for the experimental findings and should not be interpreted as a direct quantitative extrapolation to the biological activity of the whole extract. Plant extracts are complex mixtures, and their pharmacological effects may arise from additive or synergistic interactions among multiple constituents as well as from non-phenolic metabolites not included in the current docking. Accordingly, we selected a representative set of major phenolic compounds and evaluated their binding plausibility across targets aligned with our in vitro and ex vivo assays, to support a multi-target mechanistic interpretation.

#### 2.6.1. Molecular Docking

**Figure 6:**
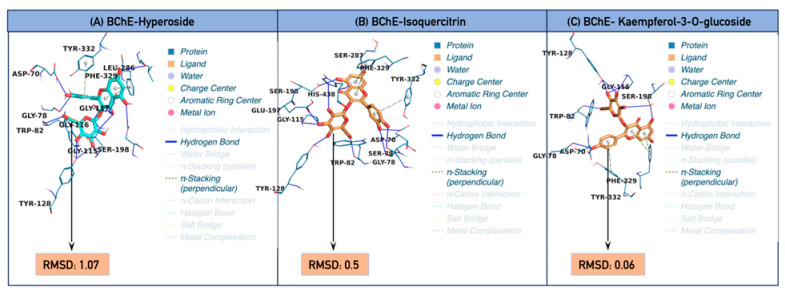
Best docking profile of *Helichrysum italicum*-derived. (**A**) BChE-Hyperoside, (**B**) BChE-Isoquercitrin, (**C**) BChE-Kaempferol-3-O-glucoside.

In this study, nine different hub phenolic compounds, 4-hydroxybenzoic acid, caffeic acid, chlorogenic acid, hyperoside, isoquercitrin, kaempferol-3-O-glucoside, quercetin, rutin, and syringic acid, were tested against multiple pharmacological targets, including AChE, BChE, α-amylase, α-glucosidase, tyrosinase, PTGS2 (COX-2), IL-6, and IL-6R. Overall results indicate that quercetin, rutin, isoquercitrin, hyperoside, and kaempferol-3-O-glucoside exhibited the strongest binding affinities for most targets, with docking scores ranging from −8.5 to −10.5 kcal·mol^−1^. The small phenolic acids 4-hydroxybenzoic acid and syringic acid exhibited weaker binding profiles, with energies mostly ranging from −4.8 to −6.6 kcal·mol^−1^. In terms of RMSD, extremely low values of 0.06–0.12 Å were observed for isoquercitrin and kaempferol-3-O-glucoside at some targets, indicating that the binding pose is quite stable and reproducible. In contrast, RMSD values exceeding 20 Å in some complexes, for example, syringic acid-PTGS2 or tyrosinase/glucosidase complexes of large glycosides, indicate unstable binding or the presence of alternative conformations. Therefore, the combination of low docking scores and low RMSDs enhances the multi-target potential of flavonoids, while small phenolic acids exhibit more limited, target-specific activity (Table S3). When target-based comparisons are examined, clear model similarities emerge. The highest binding affinities on AChE were exhibited by quercetin and chlorogenic acid. These compounds formed strong π-π interactions with critical aromatic residues, including Trp86, Tyr124, Tyr337, and Phe338; they also stabilized binding by forming hydrogen bonds with Gln71, Asp74, Gly120, Tyr133, and Glu202. Hyperoside, isoquercitrin, and kaempferol-3-O-glucoside also show a strong inhibition potential against this target with docking scores ranging from −8.5 to −8.9 kcal·mol^−1^ and low RMSD values. In the BChE profile, the best binders were hyperoside, isoquercitrin, and kaempferol-3-O-glucoside; all exhibited very strong binding at the −10.4 to −10.5 kcal·mol^−1^ level and displayed intense π interactions in the aromatic cluster involving Trp82, Gly115, and Phe329/Tyr332 (Figure 6). This suggests that flavonoid glycosides may have high selectivity for BChE. Rutin and quercetin formed the strongest bonds with α-amylase, establishing intense hydrogen bonds with central residues of the enzymatic catalytic site, such as Gln63, Arg195, Asp197, His299, and Asp300. Additionally, π-π interactions were detected with Trp58, Trp59, and Tyr62. Isoquercitrin and kaempferol-3-O-glucoside exhibited similarly strong profiles, with docking scores ranging from −8.7 to −8.9 kcal/mol. For α-glucosidase, the best-binding compounds were identified as chlorogenic acid, quercetin, isoquercitrin, kaempferol-3-O-glucoside, and especially rutin. Critical interaction points, such as Arg352, Arg334, Tyr291, and Trp331, enhanced binding. Although some glycosides exhibit high RMSD values, indicating steric constraints on entry into the active site, the interaction intensity supports their inhibitory potential. Some compounds have shown significant activity against the inflammatory targets IL-6, IL-6R, and PTGS2. Hyperoside, isoquercitrin, kaempferol-3-O-glucoside, and rutin exhibited docking scores ranging from −7.0 to −7.3 kcal/mol and formed strong hydrogen bonds with residues Pro65, Met67, Glu172, Gln175, Ser176, and Arg179 when targeting IL-6. For IL-6R, rutin, isoquercitrin, and kaempferol-3-O-glucoside had docking scores ranging from −7.4 to −8.0 kcal/mol and exhibited homologous interaction motifs with residues Arg104, Gly164, Glu277, and Gln281. For the PTGS2 (COX-2) target, rutin and quercetin had the highest binding energies at −9.9 and −9.5 kcal·mol^−1^, respectively, and showed intense interactions at the typical binding sites of COX-2 inhibitors: His207, His214, His386, His388, Tyr385, and Trp387. Isoquercitrin and hyperoside presented similar profiles with a docking score of −8.9 kcal·mol^−1^. In contrast, smaller structures, such as syringic acid, exhibited high RMSD values, indicating low binding stability. The tyrosinase target presented a different binding model compared to other enzymes. Here, glycosylated flavonoids showed a weaker binding docking score of −4.7 to −5.4 kcal·mol^−1^ and a high RMSD. Conversely, syringic acid (−6.2 kcal/mol), chlorogenic acid (−6.6 kcal/mol), and quercetin (−6.1 kcal/mol) exhibited more suitable pharmacophore profiles for tyrosinase, demonstrating lower energies and more stable positions. The key determinants of this binding are π-π stacking with His208 and hydrogen bonds formed with residues such as Glu195, Asn205, and Gly216. Overall, it was observed that most of the ligands within the scope of this study have significant multi-target activity potential. Together, quercetin, rutin, isoquercitrin, hyperoside, and kaempferol-3-O-glucoside stand out as targets for cholinesterases, carbohydrate hydrolases, and inflammation. These properties suggest that these compounds could be used as drugs to treat multiple conditions simultaneously, such as neurodegenerative diseases, diabetes, and inflammation. Upon evaluating all findings together, it is clear that the scavenging/reducing and enzyme-inhibitory capacities of the water and hydroalcoholic extracts exceed in silico predictions and are confirmed in in vitro and ex vivo models. Specifically, the reduction of the LPS-induced inflammatory response in mouse colon and liver tissues by both extracts, achieved through significant suppression of COX-2 and IL-6 gene expression levels, clearly supports the anti-inflammatory potential of these compounds. This finding aligns with docking analyses indicating that hydroxycinnamic acids (particularly chlorogenic acid) and flavonoids (quercetin, rutin, hyperoside, and isoquercitrin) exhibit robust binding to COX-2 and IL-6. Therefore, our study demonstrates that phenolic compounds consistently exhibit antioxidant and anti-inflammatory properties at the molecular binding level, in cell culture models, and in isolated tissues. We acknowledge that the interaction between quercetin and AChE has been previously investigated using docking approaches. In the present study, our objective was not to claim the first description of the AChE-quercetin, but to evaluate quercetin within an extract-driven panel of major phenolics and within a multi-target framework that includes cholinesterases, carbohydrate hydrolases, and inflammation-related targets. This integrative design allows us to connect composition-informed computational predictions with our experimental evidence, including the suppression of COX-2 and IL-6 gene expression in ex vivo tissues, and to validate selected docking poses through 100 ns MD simulations. Therefore, novelty resides in the extract-context integration and multi-target mechanistic triangulation rather than in isolated ligand–target pairs. These findings provide scientific support for the traditional use of *H. italicum* and suggest that the plant may be a potential phytotherapeutic candidate for inflammatory processes in the liver and gastrointestinal system. Future studies should expand to include in vivo pharmacodynamic models, the purification of phenolic fractions, the evaluation of their pharmacokinetic behaviour, and a more detailed analysis of their safety profiles.

#### 2.6.2. Molecular Dynamics Simulation

**Figure 7:**
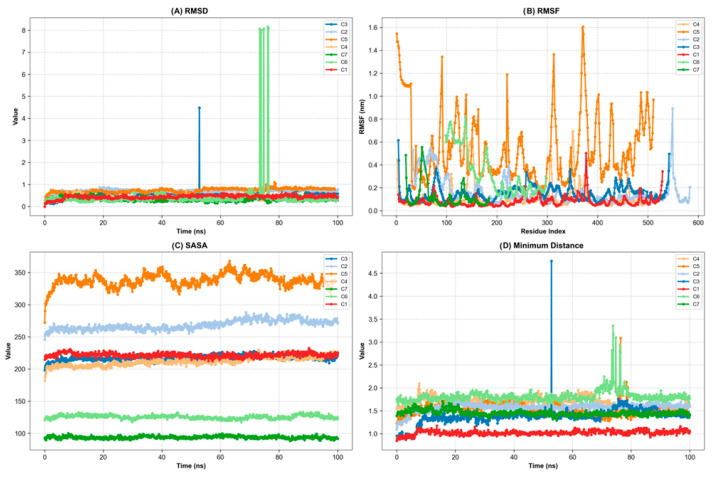
Molecular dynamics stability metrics for selected complexes: (**A**) RMSD, (**B**) RMSF, (**C**) SASA, (**D**) Minimum distance.

**Figure 8:**
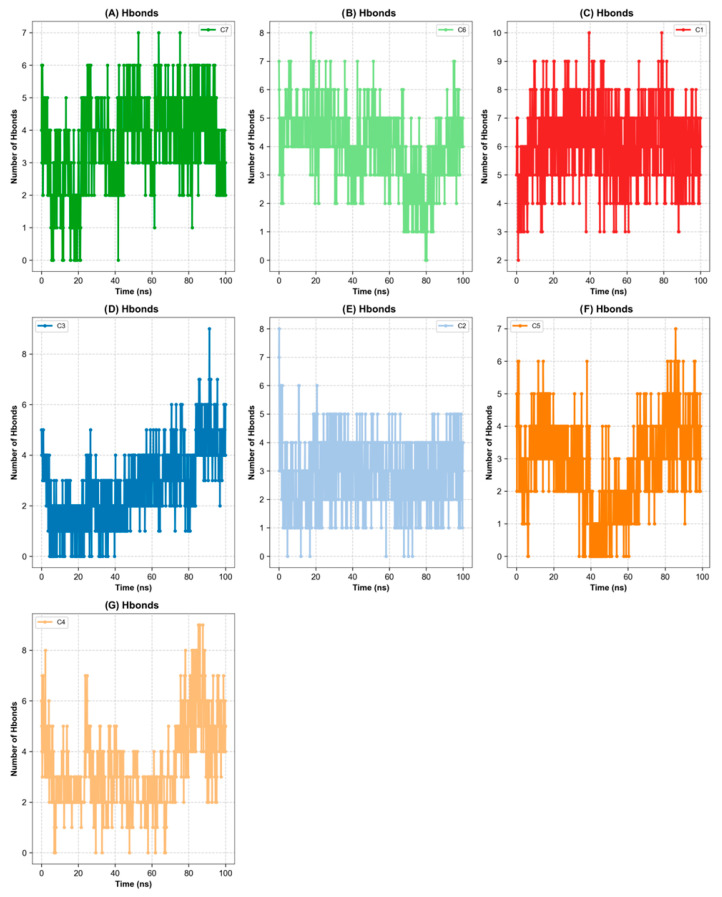
Time-resolved protein-ligand hydrogen-bond occupancy.

Molecular dynamics simulations were performed for 100 ns on seven protein-ligand complexes to evaluate the time-dependent stability of binding modes defined by docking. In this context, the C1-C7 complexes were Hyperoside-BChE, Rutin-PTGS2, Quercetin-AChE, Rutin-amylase, Rutin-glucosidase, Rutin-ILR6, and Rutin-IL6 systems, respectively. For all complexes, RMSD, RMSF (root mean square fluctuation), SASA (solvent accessible surface area), minimum ligand-protein distance, and Hbond analyses were performed to characterize the conformational stability of the binding modes multidimensionally. Overall, RMSD values ranging from 0.34 to 0.74 Å, well below the 1 Å threshold, indicate that the entire system settled into a stable conformational space throughout the simulation. Similarly, the minimum distance and H-bond profiles also support continuous contact and time-dependent interaction networks below the 3.0 Å cutoff value in all complexes (Figure 7 and Figure 8). When all these metrics are evaluated together, the C1, C2, C3, C5, C6, and C7 complexes form a highly stable cluster, with values that are quite close to one another. The average RMSDs in these complexes remained in the 0.34–0.74 Å range; particularly in C1, C6, and C7, the RMSD rapidly plateaued over time, settling around 0.4 Å. RMSF profiles indicate low-amplitude fluctuations around the binding site, suggesting that critical side chains are locked into a relatively rigid framework in the presence of the ligand. The average minimum distances remaining in the ≈1.0–1.8 Å range and contact indicate that close contact between the ligand and receptor was maintained throughout the simulation. H-bond analysis supports this picture; an average of 6 hydrogen bonds are observed at C1, 4 at C6, and 4 at C7, and most of these bonds are maintained from 0–20 ns to 80–100 ns. In the C3, the average RMSD was found to be ≈0.57 Å, the RMSF ≈ 0.15 Å, and the average minimum distance ≈ 1.38 Å; The SASA value remained at ≈217 nm^2^ and, together with an average of 3 hydrogen bonds, indicates a stable but moderately flexible interaction between the ligand and the enzyme’s binding site. In these complexes, the SASA values are also relatively low, particularly at C6, indicating that the ligands adopt a conformation embedded in the binding pocket and well protected from the solvent. The C4, while still a stable system in light of these indicators, stands out as the complex with the lowest relative score. The average RMSD value for C4 is ≈0.39 Å and the RMSF is ≈0.11 Å, placing it in the same stability band as other complexes in terms of structure; that is, there is no significant instability in terms of protein backbone and ligand conformations. However, two parameters slightly distinguish C4 from the others: (i) SASA values are higher, especially when compared to C6 and C7, suggesting that rutin is partially more exposed to the solvent in the amylase binding pocket; (ii) The Hbond profile started with a relatively low number of hydrogen bonds in the early phase of the simulation (0–20 ns), but showed a marked increase over time. Indeed, the rise in average H-bond numbers between 60–100 ns reveals that the Rutin-amylase interaction network was initially not fully organized but reorganized during the middle-late phase of the simulation, transforming into a tighter binding motif. The minimum distance profile also supports this interpretation, showing that the ligand-protein separation remained below the 3.0 Å cutoff value throughout the simulation. From this perspective, it can be said that the C4 complex is not “bad,” but rather quite stable in terms of RMSD and minimum distance. However, it is somewhat disadvantaged compared to others due to SASA and early-stage H-bond parameters. In other words, although the rutin-amylase system is structurally stable, it requires a longer adaptation period compared to other complexes for the interaction network between the ligand and the enzyme to fully establish itself (Figure 7 and Figure 8). In terms of results and recommendations, these MD simulations show that all complexes exhibit stable, well-defined conformations in the binding pocket, thereby strongly supporting the overall docking results. When all parameters are considered together, the C1, C2, C3, C5, C6, and C7 complexes are the strongest candidates due to their low RMSD/RMSF, short minimum distances, and rich, time-resistant H-bond networks. Although C4 falls slightly below this group in composite scoring, particularly due to its SASA and H-bond initial profile, it exhibits functionally significant stability, with its interaction network strengthening over time and stable RMSD/RMSF values. Therefore, while complexes in the high stability set should be prioritized in advanced stages for free energy calculations MM/PBSA, longer MD simulations, and in vitro enzyme inhibition experiments, it is recommended that the C4 complex not be overlooked, particularly in structural optimization and derivative design studies.

## 3. Materials and Methods

### 3.1. Plant Material

*Helichrysum italicum* (Roth) G.Don (Asteraceae) aerial parts were collected in the vicinity of Pescina (Abruzzo, Italy, GPS coordinates: 42°01′35″ N 13°39′32″ E) in July 2023, and then placed in a well-ventilated area to air-dry gently, away from direct sunlight, for at least three weeks. The plant material authentication was carried out by Prof. Luigi Menghini, Prof. Claudio Ferrante, and Dr. Simonetta Cristina Di Simone. During sampling, A valid specimen has been collected and is preserved in the Herbarium of the University Botanical Garden “G. d’Annunzio” “Giardino dei Semplici”. Voucher ID is HI2023-GDS1. The plant material was extracted in water and a hydroalcoholic solution. Details are included in the Supplementary Material.

### 3.2. Assays for Total Phenolics, Total Flavonoids, Antioxidant Effect and Enzyme Inhibition

The determination of total phenolic (TPC) and flavonoid (TFC) levels in the extracts and the antioxidant effects were assessed according to previously established protocols [54]. Enzyme inhibitory assays were conducted against acetylcholinesterase (AChE), butyrylcholinesterase (BChE), tyrosinase, amylase, and glucosidase using the tested extracts, according to validated protocols reported in the literature [55]. Details about the assays are included in the Supplementary Material.

### 3.3. HPLC-ESI-MS/MS Analysis of Phenolic Compounds

The qualitative and quantitative analysis of phenolics was carried out through HPLC-MS/MS, using an Agilent 1290 Infinity series and a Triple Quadrupole 6420 from Agilent Technology (Santa Clara, CA, USA) equipped with an electrospray ionization (ESI) source operating in negative and positive ionization modes, as reported in a previously published method [56]. Details about the method are fully described in the Supplementary Materials.

### 3.4. Allelopathy Assay

The potential phytotoxicity of *H. italicum* extracts was investigated through an allelopathy assay. The extracts were assayed in the concentration range 2.5–40 mg/mL. For the assay, commercial seeds of *Cichorium intybus* L. and *Dicondra repens* were selected. A detailed description of the protocol is reported in the Supplementary Material.

### 3.5. Brine Shrimp Toxicity Bioassay

The brine shrimp (*Artemia salina*) lethality assay (BSLA) was performed according to Meyer et al. (1982) and McLaughlin et al. (1998) [33,34]. Briefly, nauplii (*n* = 10) were exposed to varying concentrations (0.625–20 mg/mL) of extracts in artificial seawater (5 mL) in triplicate. Surviving nauplii were counted after 24 h, and LC50 values were calculated using GraphPad software (version 5.01). A control mortality rate of up to 10% was deemed acceptable.

### 3.6. Daphnia Magna Heart Beat Rate Assay

The tested concentration used in this study was the LC_50_ value obtained from the Brine Shrimp Lethality Assay (BSLA). For the basal condition, *Daphnia magna* were pre-treated with the extract for 15 min, after which their heartbeats were counted. To induce cardio-toxicity, a 10% EtOH solution was used. In particular, *D. magna* were pre-treated with the extract for 15 min, followed by a 2 min treatment with 10% ethanol (EtOH). After this treatment, heartbeats were counted. A negative control (H_2_O) and a positive control (10% EtOH) were performed. The experiment was performed in triplicate for each concentration and for the positive and negative controls, using different vessels for each replicate.

### 3.7. Cell Culture

The biocompatibility of *H. italicum* aqueous and hydroalcoholic extracts was also investigated on human normal colon epithelial cells CCD 841CoN (ATCC^®^ CRL-1790™) through MTT assay. The cell line was obtained from the American Type Culture Collection (ATCC, Manassas, VA, USA). Details about cell culture are described in the Supplementary Material.

### 3.8. Ex Vivo Study

The determination of *H. italicum* extracts anti-inflammatory effects was assessed on isolated colon and liver specimens from adult C57/BL6 mice (3-month-old, weight 20–25 g). Tissue samples were obtained as residual material from vehicle-treated animals randomized in previous experiments, which were approved by the local ethics committee (‘G. d’Annunzio’ University, Chieti, Italy) and Italian Health Ministry (Project no. 885/2018-PR). Colon and liver specimens were incubated with *H. italicum* aqueous and hydroalcoholic extracts (200–1000 µg/mL) for 4 h in the presence of *E. coli* LPS (50 µg/mL) and the gene expression of COX-2 and IL-6 was assessed via real-time RT-PCR. Details of the ex vivo study are included in the Supplementary Material.

### 3.9. In Silico Study

#### 3.9.1. Molecular Docking

Molecular docking was performed to evaluate the affinity for phenylethanoids and flavonoids identified in *Helichrysum italicum*. The docking study included eight therapeutic targets, namely AChE, BChE, α-amylase, α-glucosidase, IL-6, and COX-2 (PTGS2). Three-dimensional crystal structures of these enzymes have been obtained from the Protein Data Bank (PDB) (Table S2). The ligand structures have been drawn in ChemSketch (version 14.01) and energy-minimized in Avogadro v1.2.0 using the MMFF94 force field. The protein preparation was done in BIOVIA Studio and AutoDockTools v4.2.6. The ligand geometry was optimized in Avogadro v1.2.0; hydrogens were combined, and Gasteiger partial charges were assigned before docking. The docking was performed in AutoDock Vina v1.1.2 with the exhaust gas level set to 32. The binding pockets have been defined using cavity predictions from POCASA v1.1. For protocol validation, crystallized ligands were redocked into their native binding sites, and root-mean-square deviation (RMSD) values were computed to evaluate pose reproducibility [57,58]. The interaction patterns of protein ligands, such as hydrogen bonding, hydrophobic binding, and pi-stacking, have been studied using the PLIP protein ligand interaction profiler [59,60]. These steps have ensured adequate structural control and computational robustness of the docking results.

#### 3.9.2. Molecular Dynamic Simulation

Molecular dynamics (MD) simulations have been performed to evaluate the dynamic stability and binding behaviour of selected protein ligand complexes. The initial system setup was done with CHARMM GUI (Charmm-gui.org) (https://charmm-gui.org) [61,62]. The proteins and ligands were parametrically enhanced using CHARM36m [63], dissolved in a TIP3P-transparent three-point water box, and electrically neutralised by the addition of counter-ions. Bulk ionic strength was maintained at 0.15 m NaCl. Non-bonded interactions were used in Verlet’s approximation scheme, and hydrogen atoms were constrained using the LINCS linear constraint solver. Long-range electrostatics have been calculated using the Ewald PME method (Particle-Mesh Ewald). Energy minimization used the steepest descent method until the maximum force was less than 1000 kJ/mol/nm. Equilibration proceeded in two stages: first, under an NVT constant number, volume, temperature ensemble, and then under an NPT constant number, pressure, temperature ensemble at 310 K. Production trajectories were generated for 100 ns with GROMACS 2024.

### 3.10. Statistical Analysis

Data are presented as mean ± SD of 3–5 experiments performed in triplicate. Statistical analysis was conducted using analysis of variance (ANOVA) and Newman-Keuls post hoc test (GraphPad Prism 5.01), with *p* < 0.05 considered significant.

## 4. Conclusions

In conclusion, this study investigated the potential bio-pharmacological applications of water and hydroalcoholic extracts from *Helichrysum italicum* aerial parts. The extracts were obtained using an ultrasound-assisted method to mimic, albeit partially, the traditional infusion and maceration methods for preparing teas and liqueurs, respectively. Both extracts were analyzed using colorimetric and chromatographic methods, which revealed the presence of hydroxycinnamic and flavonoid phenolics, particularly in the hydroalcoholic extract. This last was also the most effective as an antioxidant and enzyme-inhibitory agent. Whilst both extracts showed similar biocompatibility limits in ecotoxicological models and non-tumoral cells in vitro. In analogy, the two extracts displayed similar potency in blunting LPS-induced increases in COX-2 and IL-6 gene expression in colon and liver tissues. This finding corroborates, at least in part, the traditional use of *H. italicum* in treating and alleviating gastrointestinal and liver disorders. In addition to these findings, the molecular docking and 100 ns molecular dynamics simulations conducted in the study demonstrated that the main phenolic components of *H. italicum* exhibit high binding affinity toward AChE, BChE, carbohydrate hydrolases, and inflammatory targets. Moreover, these compounds formed time-stable interaction networks with all the investigated proteins. Therefore, the inhibitory effects of the extracts on COX-2 and IL-6 gene expression are supported by a rational molecular-level binding model. Importantly, the combined application of chemical, biological, and computational approaches offers a robust and translational framework that enhances the reliability of the results and facilitates the identification of plant-based compounds with genuine therapeutic promise. Overall, these findings underscore the need for further research, including in vivo studies, to thoroughly evaluate the extracts’ pharma-toxicological profile using diverse analytical and pharmacological models.

## Figures and Tables

**Table 1:** Total phenolic (TPC) and flavonoid (TFC) levels of *H. italicum* extracts.

Solvents	TPC (mg GAE/g Extract)	TFC (mg RE/g Extract)
Ethanol 50%	86.59 ± 1.31 ^a^	62.05 ± 0.42 ^a^
Water	65.95 ± 0.59 ^b^	11.17 ± 0.09 ^b^

Values are reported as mean ± SD of three parallel experiments. TPC: Total phenolic content; TFC: Total flavonoid content; GAE: Gallic acid equivalent; RE: Rutin equivalent. Different letters indicate significant differences in the tested extracts (*p* < 0.05).

**Table 2:** Phenolic profile of the water and hydroalcoholic (ethanol 70%) *Helicrhysum italicum* extracts (mg/kg dry extract). Hydroxycinnamic acids and quercetin derivatives were found as the main phytochemicals.

N°	Chemicals	Ethanol 50%	Water
1	Gallic acid	12.90	6.41
2	Neochlorogenic acid	1007.51	1167.45
3	Delphinidin-3-galactoside	n.d.	n.d.
4	Catechin	n.d.	n.d.
5	Procyanidin B2	n.d.	n.d.
6	Chlorogenic acid	6468.40	2265.72
7	4-Hydroxy benzoic acid	126.57	145.72
8	Epicatechin	n.d.	n.d.
9	Cyanidin-3-glucoside	21.23	n.d.
10	Petunidin-3-glucoside	n.d.	n.d.
11	3-Hydroxy benzoic acid	n.d.	n.d.
12	Caffeic acid	n.d.	4684.33
13	Vanillic acid	n.d.	219.46
14	Resveratrol	n.d.	n.d.
15	Pelargonidin-3-glucoside	n.d.	n.d.
16	Pelargonidin-3-rutinoside	n.d.	n.d.
17	Malvidin-3-galactoside	n.d.	n.d.
18	Syringic acid	138.25	140.68
19	Procyanidin A2	n.d.	n.d.
20	P-Coumaric acid	47.26	380.93
21	Ferulic acid	86.46	103.30
22	3,5-Dicaffeoylquinic acid	n.d.	n.d.
23	Rutin	18,260.35	n.d.
24	Hyperoside	2410.78	259.59
25	Isoquercitrin	2269.97	150.71
26	Delphinidin-3,5-diglucoside	2456.08	164.54
27	Phloridzin	5.64	n.d.
28	Quercitrin	n.d.	n.d.
29	Myricetin	n.d.	n.d.
30	Naringin	n.d.	n.d.
31	Kaempferol-3-glucoside	205.87	5.47
32	Hesperidin	n.d.	n.d.
33	Ellagic acid	n.d.	n.d.
34	trans-cinnamic acid	n.d.	n.d.
35	Quercetin	973.97	38.34
36	Phloretin	n.d.	n.d.
37	Kaempferol	97.17	n.d.
38	Isorhamnetin	61.54	7.90
	Total phenolic content	34,649.93	9740.53

**Table 3:** Antioxidant properties of *H. italicum* extracts.

Solvents	DPPH (mg TE/g Extract)	ABTS (mg TE/g Extract)	CUPRAC (mg TE/g Extract)	FRAP (mg TE/g Extract)	MCA (mg EDTAE/g Extract)	PBD (mmol TE/g Extract)
Ethanol 50%	271.44 ± 2.90 ^a^	310.52 ± 5.47 ^a^	464.49 ± 21.62 ^a^	257.24 ± 7.29 ^a^	16.86 ± 0.52 ^a^	3.35 ± 0.14 ^a^
Water	130.14 ± 1.78 ^b^	113.46 ± 5.77 ^b^	269.51 ± 2.76 ^b^	167.77 ± 2.86 ^b^	12.73 ± 1.02 ^b^	2.50 ± 0.09 ^b^

Values are reported as mean ± SD of three parallel measurements. TE: Trolox equivalent; EDTAE: EDTA equivalent. Different letters indicate significant differences in the tested extracts (*p* < 0.05). ABTS, 2,2′-azino-bis(3-ethylbenzothiazoline) 6-sulfonic acid; CUPRAC, cupric ion reducing antioxidant capacity; DPPH, 1,1-diphenyl-2-picrylhydrazyl; FRAP, ferric ion reducing antioxidant power; MCA, metal chelating activity; PBD, phosphomolybdenum activity.

**Table 4:** Enzyme inhibitory of *H. italicum* extracts.

Solvents	AChE (mg GALAE/g Extract)	BChE (mg GALAE/g Extract)	Tyrosinase (mg KAE/g Extract)	Amylase (mmol ACAE/g Extract)	Glucosidase (mmol ACAE/g Extract)
Ethanol 50%	2.18 ± 0.01 ^a^	0.50 ± 0.01 ^b^	45.51 ± 3.36 ^a^	0.33 ± 0.02 ^a^	0.84 ± 0.01 ^a^
Water	1.26 ± 0.07 ^b^	1.11 ± 0.16 ^a^	29.00 ± 0.54 ^b^	0.05 ± 0.002 ^b^	0.68 ± 0.03 ^b^

Values are reported as mean ± SD of three parallel measurements. GALAE: Galantamine; KAE: Kojic acid; ACAE: Acarbose equivalent; na: not active. Different letters indicate significant differences in the tested extracts (*p* < 0.05). AChE: Acetylcholinesterase; BChE: Butrylcholinesterase.

**Table 5:** Brine shrimp lethality assay results in terms of LC_50_ value and toxicity levels according to Meyer’s and Clarckson’s classifications. Tested samples: *Helichrysum italicum* water and hydroalcoholic extracts, in a concentration range between 0.625 and 20 mg/mL.

Tested Compound	Concentration Range [mg/mL]	LC_50_ (mg/mL)	95% Confidence Interval	R^2^	Toxicity Class	
					Meyer’s Classification	Clarkson’s Classification
Water	[0.625–20]	2.84	2.26–3.56	0.98	non-toxic	non-toxic
Ethanol 50%	[0.625–20]	5.07	3.46–7.43	0.96	non-toxic	non-toxic

## Data Availability

The data that support the findings of this study are available from the corresponding author upon reasonable request.

## Supplementary Materials

The following supporting information can be downloaded at: https://www.mdpi.com/article/10.3390/plants15071108/s1, Table S1: HPLC-MS/MS acquisition parameters (dynamic-MRM mode) used for the analysis of the 38 markers. Table S2: Docking grid center coordinates and box dimensions for the selected protein targets. Table S3: Docking scores (kcal/mol) and key interacting residues of the ligand–protein complexes. Detailed descriptions of: Reagents and Standards; Assays for Total phenolics, Total flavonoids, Antioxidant effect and Enzyme inhibition; HPLC-ESI-MS/MS Analysis of Phenolic Compounds; Allelopathy Assay; Cell Culture; Ex Vivo Study [55,56,64,65,66].

## Abbreviations

ABTS	2,2′-azinobis-(3-ethylbenzothiazoline-6-sulfonic acid)
AChE	acetylcholinesterase
BChE	butyrylcholinesterase
COX-2	cyclooxygenase-2
CUPRAC	CUPric reducing antioxidant capacity
DPPH	1,1-diphenyl-2-picrylhydrazil
FRAP	ferric reducing antioxidant power
GAE	gallic acid equivalents
Interleukin-6	IL-6
LPS	lipopolysaccharide
MD	Molecular dynamics
MTT	3-(4,5-dimethylthiazol-2-yl)-2,5-diphenyltetrazolium bromide
RE	rutin equivalents
RMSD	root mean square deviation
RMSF	root mean square fluctuation
SASA	solvent accessible surface area
TE	Trolox equivalents
TFC	total flavonoid content
TPC	total phenolic content
