# Supplementary material for: The Green Treasure from Appennine Flora for Colon and Liver Health: Characterization and Evaluation of the Protective Effects from Aerial Parts of Helichrysum italicum
Source: Plants (Basel). 2026 Apr 3;15(7):1108. doi: 10.3390/plants15071108 (PMC13075095; doi:10.3390/plants15071108)
Supplement: Supplementary file 1 [file plants-15-01108-s001.zip › plants-4089833-supplementary.pdf]

Supplementary Material

The green treasure from Appennine flora for colon and liver health: characterization and evaluation of the protective effects from aerial parts of *Helichrysum italicum*

Maria Loreta Libero[1], Gianluca Genovesi[1**], Mariachiara Gabriele[1], Annalisa Chiavaroli[1], Giustino Orlando[1], Luigi Brunetti[1], Sheila Leone[1], Lucia Recinella[1], Gokhan Zengin[2], Giovanni Caprioli[3], Laura Acquaticci[3], Mehmet Veysi Cetiz[4], Luigi Menghini[1], Claudio Ferrante[1*], Simonetta Cristina Di Simone[1]**

[1]Botanical Garden "Giardino dei Semplici", Department of Pharmacy, "G. d'Annunzio" University "Chieti-Pescara", Via dei Vestini n. 31, 66100 Chieti, Italy: maria.libero@unich.it (M. Libero); gianluca.genovese@phd.unich.it (G. Genovese); mariachiara.gabriele@phd.unich.it (M. Gabriele); annalisa.chiavaroli@unich.it (A. Chiavaroli); giustino.orlando@unich.it (G. Orlando); luigi.brunetti@unich.it (L. Brunetti); sheila.leone@unich.it (S. Leone); lucia.recinella@unich.it (L. Recinella); luigi.menghini@unich.it (L. Menghini); claudio.ferrante@unich.it (C. Ferrante); simonetta.disimone@unich.it (S.C. Di Simone).

[2]Physiology and Biochemistry Research Laboratory, Department of Biology, Science Faculty, Selcuk University, Konya, 42130, Turkey: gokhanzengin@selcuk.edu.tr (G. Zengin).

[3]Chemistry Interdisciplinary Project (CHIP) research center, School of Pharmacy, University of Camerino, 62032 Camerino, Italy: giovanni.caprioli@unicam.it (G. Caprioli); laura.acquaticci@unicam.it (L. Acquaticci).

[4]Department of Medicinal Biochemistry, Faculty of Medicine, Harran University, Sanliurfa, Turkey: mvcetiz@gmail.com (M.V. Cetiz).

* Correspondence: Prof. Claudio Ferrante (claudio.ferrante@unich.it)

** These authors equally contributed to the manuscript.

Assays for Total Phenolic and Flavonoid Contents

The total phenolic content was determined by employing the methods given in the literature with some modification. Sample solution (0.25 mL) was mixed with diluted Folin–Ciocalteu reagent (1 mL, 1:9, v/v) and shaken vigorously. After 3 min, Na_2CO_3 solution (0.75 mL, 1%) was added and the sample absorbance was read at 760 nm after a 2 h incubation at room temperature. The total phenolic content was expressed as milligrams of gallic acid equivalents (mg GAE/g extract)(Uysal et al., 2017).

The total flavonoid content was determined using the $AlCl_3$ method. Briefly, sample solution (1 mL) was mixed with the same volume of aluminum trichloride (2%) in methanol. Similarly, a blank was prepared by adding sample solution (1 mL) to methanol (1 mL) without $AlCl_3$. The sample and blank absorbances were read at 415 nm after a 10 min incubation at room temperature. The absorbance of the blank was subtracted from that of the sample. Rutin was used as a reference standard and the total flavonoid content was expressed as milligrams of rutin equivalents (mg RE/g extract) (Uysal et al., 2017).

Determination of Antioxidant and Enzyme Inhibitory Effects

Antioxidant (DPPH and ABTS radical scavenging, reducing power (CUPRAC and FRAP), phosphomolybdenum and metal chelating (ferrozine method)) and enzyme inhibitory activities (cholinesterase (Elmann's method), tyrosinase (dopachrome method), α-amylase (iodine/potassium iodide method), α -glucosidase (chromogenic PNPG method) and pancreatic lipase (p-nitrophenyl butyrate (p-NPB)

method) were determined using the methods previously described by Uysal et al. (Uysal et al., 2017) and Grochowski et al. (Grochowski et al., 2017)

For the DPPH (1,1-diphenyl-2-picrylhydrazyl) radical scavenging assay: Sample solution was added to 4 mL of a 0.004% methanol solution of DPPH. The sample absorbance was read at 517 nm after a 30 min incubation at room temperature in the dark. DPPH radical scavenging activity was expressed as milligrams of trolox equivalents (mg TE/g extract).

For ABTS (2,2′-azino-bis(3-ethylbenzothiazoline) 6-sulfonic acid) radical scavenging assay: Briefly, ABTS+ was produced directly by reacting 7 mM ABTS solution with 2.45 mM potassium persulfate and allowing the mixture to stand for 12–16 in the dark at room temperature. Prior to beginning the assay, ABTS solution was diluted with methanol to an absorbance of 0.700 ± 0.02 at 734 nm. Sample solution was added to ABTS solution (2 mL) and mixed. The sample absorbance was read at 734 nm after a 30 min incubation at room temperature. The ABTS radical scavenging activity was expressed as milligrams of trolox equivalents (mg TE/g extract).

For CUPRAC (cupric ion reducing activity) activity assay: Sample solution was added to premixed reaction mixture containing $CuCl_2$ (1 mL, 10 mM), neocuproine (1 mL, 7.5 mM) and NH_4Ac buffer (1 mL, 1 M, pH 7.0). Similarly, a blank was prepared by adding sample solution (0.5 mL) to premixed reaction mixture (3 mL) without $CuCl_2$. Then, the sample and blank absorbances were read at 450 nm after a 30 min incubation at room temperature. The absorbance of the blank was subtracted from that of the

sample. CUPRAC activity was expressed as milligrams of trolox equivalents (mg TE/g extract).

For FRAP (ferric reducing antioxidant power) activity assay: Sample solution was added to premixed FRAP reagent (2 mL) containing acetate buffer (0.3 M, pH 3.6), 2,4,6-tris(2-pyridyl)-S-triazine (TPTZ) (10 mM) in 40 mM HCl and ferric chloride (20 mM) in a ratio of 10:1:1 (v/v/v). Then, the sample absorbance was read at 593 nm after a 30 min incubation at room temperature. FRAP activity was expressed as milligrams of trolox equivalents (mg TE/g extract).

For phosphomolybdenum method: Sample solution was combined with 3 mL of reagent solution (0.6 M sulfuric acid, 28 mM sodium phosphate and 4 mM ammonium molybdate). The sample absorbance was read at 695 nm after a 90 min incubation at 95 °C. The total antioxidant capacity was expressed as millimoles of trolox equivalents (mmol TE/g extract).

For metal chelating activity assay: Briefly, sample solution was added to $FeCl_2$ solution (0.05 mL, 2 mM). The reaction was initiated by the addition of 5 mM ferrozine (0.2 mL). Similarly, a blank was prepared by adding sample solution (2 mL) to $FeCl_2$ solution (0.05 mL, 2 mM) and water (0.2 mL) without ferrozine. Then, the sample and blank absorbances were read at 562 nm after 10 min incubation at room temperature. The absorbance of the blank was sub-tracted from that of the sample. The metal chelating activity was expressed as milligrams of EDTA (disodium edetate) equivalents (mg EDTAE/g extract).

For Cholinesterase (ChE) inhibitory activity assay: Sample solution (was mixed with DTNB (5,5-dithio-bis(2-nitrobenzoic) acid, Sigma, St. Louis, MO, USA) (125 μL) and AChE (acetylcholines-terase (Electric ell acetylcholinesterase, Type-VI-S, EC 3.1.1.7,Sigma)), or BChE (butyrylcholinesterase (horse serum butyrylcholinesterase, EC 3.1.1.8, Sigma)) solution (25 μL) in Tris–HCl buffer (pH 8.0) in a 96-well microplate and incubated for 15 min at 25 °C. The reaction was then initiated with the addition of acetylthiocholine iodide (ATCI, Sigma) or butyrylthiocholine chloride (BTCl, Sigma) (25 μL). Similarly, a blank was prepared by adding sample solution to all reaction reagents without enzyme (AChE or BChE) solution. The sample and blank absorbances were read at 405 nm after 10 min incubation at 25 °C. The absorbance of the blank was subtracted from that of the sample and the cholinesterase inhibitory activity was expressed as galanthamine equivalents (mgGALAE/g extract).

For Tyrosinase inhibitory activity assay: Sample solution was mixed with tyrosinase solution (40 μL, Sigma) and phosphate buffer (100 μL, pH 6.8) in a 96-well microplate and incubated for 15 min at 25 °C. The reaction was then initiated with the addition of L-DOPA (40 μL, Sigma). Similarly, a blank was prepared by adding sample solution to all reaction reagents without enzyme (tyrosinase) solution. The sample and blank absorbances were read at 492 nm after a 10 min incubation at 25 °C. The absorbance of the blank was subtracted from that of the sample and the tyrosinase inhibitory activity was expressed as kojic acid equivalents (mgKAE/g extract).

For α-amylase inhibitory activity assay: Sample solution was mixed with α-amylase solution (ex-porcine pancreas, EC 3.2.1.1, Sigma) (50 μL) in phosphate buffer (pH 6.9 with 6 mM sodium chloride) in a 96-well microplate and incubated for 10 min at 37 °C. After pre-incubation, the reaction was initiated with the addition of starch solution (50 μL, 0.05%). Similarly, a blank was prepared by adding sample solution to all reaction reagents without enzyme (α-amylase) solution. The reaction mixture was incubated 10 min at 37 °C. The reaction was then stopped with the addition of HCl (25 μL, 1 M). This was followed by addition of the iodine-potassium iodide solution (100 μL). The sample and blank absorbances were read at 630 nm. The absorbance of the blank was subtracted from that of the sample and the α-amylase inhibitory activity was expressed as acarbose equivalents (mmol ACE/g extract).

For α-glucosidase inhibitory activity assay: Sample solution was mixed with glutathione (50 μL), α-glucosidase solution (from Saccharomyces cerevisiae, EC 3.2.1.20, Sigma) (50 μL) in phosphate buffer (pH 6.8) and PNPG (4-N-trophenyl-α-D-glucopyranoside, Sigma) (50 μL) in a 96-well microplate and incubated for 15 min at 37 °C. Similarly, a blank was prepared by adding sample solution to all reaction reagents without enzyme (α-glucosidase) solution. The reaction was then stopped with the addition of sodium carbonate (50 μL, 0.2 M). The sample and blank absorbances were read at 400 nm. The absorbance of the blank was subtracted from that of the sample and the α-glucosidase inhibitory activity was expressed as acarbose equivalents (mmol ACE/g extract).

HPLC-ESI-MS/MS Analysis of Phenolic Compounds

HPLC-MS/MS studies were performed using an Agilent 1290 Infinity series and a Triple Quadrupole 6420 from Agilent Technology (Santa Clara, CA) equipped with an electrospray ionization (ESI) source operating in negative and positive ionization modes by following a previous published method (Mustafa et al., 2022). The separation of target compounds was achieved on a Synergi Polar-RP C18 analytical column (250 mm x 4.6 mm, 4 μm). The mobile phase was a mixture of (A) water and (B) methanol, both with formic acid 0.1%, at a flow rate of 0.8 mL min-1 in gradient elution mode, the injection volume was 2 μL and the temperature of the column was 30 °C. In the source of ionization the temperature of the drying gas in the ionization source was 350 °C, the gas flow was 12 L/min, the nebulizer pressure was 55 psi, and the capillary voltage was 4000 V. Detection was performed in the dynamic-multiple reaction monitoring (dynamic-MRM) mode, and the dynamic-MRM peak areas were integrated for quantification (Table S1).

No.	Compounds	Precursor ion, *m/z*	Product ion, *m/z*	Fragm-entor, V	Collision energy, V	Polarity	Retention time (Rt, min)	Delta retention time (ΔRt)
1	Gallic acid	169	125.2*	97	12	Negative	6.96	2
2	Neochlorogenic acid	353	191.2*, 179	82	12, 12	Negative	9.52	2
3	Delphinidin-3-galactoside	465.01	303*	121	20	Positive	11.36	2
4	(+)-Catechin	289	245.2*,109.2	131	8, 20	Negative	11.44	2
5	Procyanidin B2	576.99	576.99*, 321.2	160	0, 32	Negative	12.41	2
6	Chlorogenic acid	353	191.2*, 127.5	82	12, 20	Negative	12.42	2
7	*p*-Hydroxybenzoic acid	137	93.2*	92	16	Negative	12.86	2
8	(-)-Epicatechin	289	245.1*, 109.1	126	8, 20	Negative	13.03	2
9	Cyanidin-3-glucoside	449	287.3*, 255.6	121	20, 20	Positive	13.14	2
10	Petunidin-3-glucoside	479.01	317*, 302	121	20, 44	Positive	13.26	2
11	3-Hydroxybenzoic acid	137	93.2*	88	8	Negative	13.59	2
12	Caffeic acid	179	135.2*, 134.1	92	12, 24	Negative	13.65	2
13	Vanillic acid	167	152.4*, 108.1	88	12, 20	Negative	14.32	2
14	Resveratrol	227	185*	131	12	Negative	14.40	2
15	Pelargonidin-3-glucoside	433.01	271*, 121	116	24, 50	Positive	14.52	2
16	Pelargonidin-3-rutinoside	579.01	271*	145	32	Positive	14.56	2
17	Malvidin-3-galactoside	493.01	331*, 315.1	121	20, 50	Positive	14.64	2
18	Syringic acid	196.9	182.2*, 121.2	93	8, 12	Negative	15.28	2
19	Procyanidin A2	575	575*, 285	170	0, 20	Negative	16.18	2
20	*p*-Coumaric acid	163	119.2*, 93.2	83	12, 36	Negative	16.70	2
21	Ferulic acid	193	134.2*, 131.6	83	12, 8	Negative	17.10	2
22	3,5-Dicaffeoylquinic acid	514.9	353.1*, 191	117	8, 28	Negative	17.61	2
23	Rutin	609	300.2*, 271.2	170	32, 50	Negative	17.73	2
24	Hyperoside	465.01	303*, 61.1	97	8, 50	Positive	18.33	2
25	Isoquercitrin	463	271.2*, 300.2	155	44, 24	Negative	18.36	2
26	Delphinidin-3,5-diglucoside	462.9	300.1*	165	24	Negative	18.38	2
27	Phloridzin	435.39	273*, 167	155	8, 28	Negative	18.83	2
28	Quercitrin	446.99	300.2*, 301.2	160	24, 16	Negative	19.61	2
29	Myricetin	316.99	179.1*, 182	150	16, 24	Negative	19.61	2
30	Naringin	578.99	271.3*, 151.3	170	32, 44	Negative	19.62	2
31	Kaempferol-3-glucoside	447	284.2*, 255.2	170	24, 40	Negative	19.77	2
32	Hesperidin	611.01	303*, 334.8	112	20, 12	Positive	20.19	2
33	Ellagic acid	301	301*, 229	170	0, 24	Negative	21.41	2
34	*trans*-cinnamic acid	149	131.2	74	4	Positive	21.44	2
35	Quercetin	300.99	151.2*, 179.2	145	16, 12	Negative	21.87	2
36	Phloretin	272.99	167*, 123	116	8, 20	Negative	22.30	2
37	Kaempferol	287.01	153*, 69.1	60	36, 50	Positive	23.84	2
38	Isorhamnetin	314.99	300.2*, 196.1	145	16, 4	Negative	24.57	2

Table S1. HPLC–MS/MS acquisition parameters (dynamic-MRM mode) used for the analysis of the 38 marker compounds.

* These product ions were used for quantification.

Allelopathy Assay

A Petri dish-based experiment was carried out to evaluate the potential phytotoxic activity of *H. italicum* water and hydroalcoholic extracts in a concentration range from 40 to 2.5 mg/mL. Commercial seeds of *Cichorium intybus* L. and *Dicondra repens* were selected for the test due to their fast germination rate and sensitivity. The producers guarantee a germination rate of the seeds >70%, also confirmed by preliminary germination tests. The assay was conducted in 90 mm Petri dishes, each containing a double layered filter paper disk, previously soaked with 3 mL of the plant extract at the different concentrations. Distilled water was used as negative control. The seeds of *C. intybus* and *D. repens* were surface-sterilized using a diluted (NaClO:dH2O, 1:9) commercial bleaching liquid for 10 minutes. After thoroughly rinsing the seeds with sterile distilled water to remove any traces of bleach, 10 seeds of the corresponding seed variety were arranged on the filter paper disks, previously divided into 2 distinct areas. Petri dishes were then sealed with parafilm, to ensure a closed-system model and incubated in darkness at room temperature (25±2°C) for 96h. The criterion for determining germination was the emergence of a radicle at least 2 mm in length, exhibiting typical geotropic curvature. Seeds that only swelled but did not fully germinate were excluded. Radicle length was measured and categorized by elongation rate: low (<0.4 cm), medium (0.5-0.9 cm), or high (>1 cm). The number of germinated seeds and their radicle lengths were recorded after 96 hours, and results were reported as mean Germination Percentage and Growth Percentage, that is the mean of seedlings lenght compared to control samples. Experiments were conducted in triplicate.

Cell Culture

The human normal colon epithelial cell line CCD 841CoN (ATCC® CRL-1790™) was obtained from the American Type Culture Collection (ATCC, Manassas, VA, USA). Cells were maintained in Eagle's Minimum Essential Medium (EMEM, ATCC® 30-2003™, Manassas, VA, USA) supplemented with 10% heat-inactivated fetal bovine

serum (FBS, ATCC® 30-2020™ Manassas, VA, USA). Cultures were incubated at 37 °C in a humidified atmosphere with 5% CO_2. Medium was changed twice per week, and cells were sub-cultured at 80-90% confluence using 0.25% trypsin-EDTA solution (ATCC® 30-2101™, Manassas, VA). Upon reaching adequate confluence, cells were passaged and seeded for subsequent experiments. Cells were seeded in 96-well plates at a density of 5×10^4 cells/well and allowed to adhere for 24 h under standard culture conditions (37 °C, 5% CO_2, humidified atmosphere). After 24 hours, cells were treated with either control (Ctrl: culture medium) or water or hydroalcoholic extracts at different concentrations (7.8-1000 μg/mL) and incubated overnight. Thereafter, 20 μL of 3-(4,5-dimethylthiazol-2-yl)-2,5-diphenyltetrazolium bromide (MTT: 5 mg/mL in PBS) was added to each well, and the plate was incubated for 3 h at 37 °C. After that, the formed formazan dye was solubilised with dimethyl sulfoxide, and the cells were incubated in the dark room on a plate shaker at 200 rpm for 3 h (37 °C). The absorbance was measured at λ = 540 nm by a multi-plate reader. Effects on cell viability were compared to the untreated control group (Ctrl).

Ex Vivo Study

Adult C57/BL6 mice (3-month-old, weight 20-25 g) were housed in Plexiglas cages (2-4 animals per cage; 55 cm × 33 cm × 19 cm) and maintained under standard laboratory conditions (21 ± 2 °C; 55 ± 5% humidity) on a 14/10 h light/dark cycle, with ad libitum access to water and food. Housing conditions and experimentation procedures were strictly in agreement with the European Community ethical regulations (EU Directive no. 63/2010) on the care of animals for scientific research. According to the recognized principles of "Replacement, Refinement and Reduction in Animals in Research", colon and liver specimens were obtained as residual material from vehicle-treated animals randomized in our previous experiments, approved by the local ethical committee ('G. d'Annunzio' University, Chieti, Italy) and Italian Health Ministry (Project no. 885/2018-PR). The fragments of colon and liver tissue were collected from euthanized mice, were maintained in an humidified incubator with 5% CO_2 at 37°C for 4 h

(incubation period), in RPMI buffer with added bacterial LPS (50 μg/mL) and in the presence of water and hydroalcoholic extracts of *H. italicum* (200-1000 μg/mL). In mouse and liver colon, the gene expression of COX-2 and IL-6 was conducted as previously reported (Orlando et al., 2021). Briefly, total RNA was extracted from colon specimens using TRI Reagent (Sigma-Aldrich, St. Louis, MO). Contaminating DNA was be removed using 2 units of RNase-free DNase 1 (DNA-free kit, Ambion, Austin, TX). The RNA concentration was quantified at 260 nm by spectrophotometer reading (BioPhotometer, Eppendorf, Hamburg, Germany) and its purity was assessed by the ratio at 260 and 280 nm readings. The quality of the extracted RNA samples was also determined by electrophoresis through agarose gels and staining with ethidium bromide, under UV light. One microgram of total RNA extracted from each sample in a 20 μL reaction volume was reverse transcribed using a High Capacity cDNA Reverse Transcription Kit (Thermo Fisher Scientific Inc., Monza, Italy). Reactions will be incubated in a 2720 Thermal Cycler (Thermo Fisher Scientific Inc.) initially at 25 °C for 10 min, then at 37 °C for 120 min, and finally at 85 °C for 5 s. Gene expression was determined by quantitative real-time PCR using TaqMan probe-based chemistry. PCR primers and TaqMan probes, including β-actin used as the housekeeping gene, were purchased from Thermo Fisher Scientific Inc. The real-time PCR was carried out in triplicate for each cDNA sample in relation to each of the investigated genes. Data will be elaborated with the Sequence Detection System (SDS) software version 2.3 (Thermo Fisher Scientific Inc.). Gene expression will be relatively quantified by the comparative $2^{-\Delta\Delta Ct}$ method (Livak and Schmittgen, 2001).

Table S2: Docking grid center coordinates and box dimensions for the selected protein targets.

TARGET PROTEIN	*PDB ID*	*CENTER X, Y, Z*	*GRID SIZE X, Y, Z*
**ACHE**	7E3H	-54.44, 32.90, -28.65	25, 25, 25
**BCHE**	6EQP	32.16, -16.33, 40.73	25, 25, 25
**AMYLASE**	2QV4	14.188, 48.964, 22.886	28, 28, 24
**GLUCOSIDASE**	7KBJ	-18.79, 2.18, 15.75	25, 25, 25
**TYROSINASE**	6QXD	21.81, 12.22, 91.40	25, 25, 25
**IL-6**	5FUC	-56.346, -13.446, 7.413	28, 38, 48
**IL-6R**	5FUC	-28.434, 19.999, 44.433	40, 40, 60
**PTGS2**	5F19	23.467, 43.43, 59.37	110, 126, 110

Table S3: Docking scores (kcal/mol) and key interacting residues of the ligand–protein complexes.

Compound	Target	PDB ID	Binding energy	RMSD	Type and Binding site
4- hydroxy benzoic acid	AChE	7E3H	-6.4	0.6	Hbond: TYR:337<br>Hydrophobic: TRP:86;TYR:337<br>Pi-stacking: TRP:86;TRP:86
Caffeic acid	AChE	7E3H	-7.4	0.9	Hbond: ASP:74;GLY:120;TYR:124;TYR:133;TYR:133;TYR:337<br>Hydrophobic: TRP:86;TRP:86<br>Pi-stacking: TRP:86
Chlorogenic acid	AChE	7E3H	-9.0	1.1	Hbond: GLY:120;TYR:124;TYR:124;TYR:133;GLU:202;TYR:337<br>Hydrophobic: TRP:86;TRP:86;PHE:338;TYR:341;TYR:341
Hyperoside	AChE	7E3H	-8.9	0.7	Hbond: ASP:74;GLY:120;GLY:120;GLY:121;TYR:124;SER:125;GLY:126;TYR:133;TYR:133;GLU:202;PHE:295<br>Hydrophobic: TRP:86;TYR:337;PHE:338<br>Pi-stacking: TYR:341
Isoquercitrin	AChE	7E3H	-8.7	0.1	Hbond: ASP:74;GLY:120;GLY:120;GLY:121;TYR:124;SER:125;GLY:126;GLU:202;SER:203;PHE:295<br>Hydrophobic: TRP:86;TYR:337;PHE:338;TYR:341
Kaempferol-3-O-glucoside	AChE	7E3H	-8.5	1.0	Hbond: ASP:74;GLY:120;TYR:124;TYR:124;SER:125;SER:125;GLY:126;TYR:133;PHE:295;HIS:447<br>Hydrophobic: TRP:86;TYR:337;PHE:338;TYR:341
Quercetin	AChE	7E3H	-9.8	0.9	Hbond: GLN:71;ASP:74;GLY:120;TYR:133;GLU:202;TYR:337<br>Hydrophobic: ASP:74;TRP:86<br>Pi-stacking: TRP:86;TRP:86;TYR:124
Rutin	AChE	7E3H	-5.0	1.1	Hbond: GLN:71;ASN:87;GLY:120;GLY:121;TYR:124;TYR:124;GLY:126;GLU:202;TYR:337<br>Hydrophobic: TRP:86;TYR:124;TYR:124;PHE:297;PHE:338;PHE:338;TYR:341
Syringic acid	AChE	7E3H	-6.6	3.3	Hbond: TYR:124;TYR:124;TYR:337<br>Hydrophobic: TRP:86;TYR:337

					Pi-stacking: TRP:86<br>Salt bridge: HIS:447
4- hydroxy benzoic acid	Amylase	2qv4	-5.5	0.8	Hbond: ASP:300<br>Hydrophobic: TRP:58;TRP:59;TYR:62;TYR:62;LEU:165<br>Salt bridge: ARG:195;HIS:299
Caffeic acid	Amylase	2qv4	-6.5	1.1	Hbond: GLN:63;THR:163;ARG:195;ARG:195;ASP:197;ALA:198;HIS:299;ASP:300<br>Hydrophobic: TYR:62;TYR:62
Chlorogenic acid	Amylase	2qv4	-7.9	0.9	Hbond: GLN:63;GLN:63;THR:163;ASP:197;ASP:300<br>Hydrophobic: TRP:59;TRP:59<br>Pi-stacking: TRP:59<br>Salt bridge: ARG:195;HIS:299;HIS:305
Hyperoside	Amylase	2qv4	-8.6	0.8	Hbond: ARG:195;ARG:195;LYS:200;HIS:201;HIS:299;ASP:300;ASP:300;ASP:300;HIS:305
Isoquercitrin	Amylase	2qv4	-8.9	0.1	Hbond: GLN:63;GLU:233;HIS:299;ASP:300;HIS:305<br>Hydrophobic: TRP:58;TRP:59<br>Pi-stacking: TRP:59<br>Salt bridge: HIS:305
Kaempferol-3-O-glucoside	Amylase	2qv4	-8.7	1.0	Hbond: GLN:63;GLN:63;ARG:195;ASP:300;ASP:300;HIS:305<br>Hydrophobic: TRP:58;TRP:59;TYR:62<br>Pi-stacking: TRP:59<br>Salt bridge: HIS:305
Quercetin	Amylase	2qv4	-9.2	0.5	Hbond: GLN:63;ARG:195;ASP:197;HIS:299;ASP:300<br>Hydrophobic: TRP:58;TYR:62;TYR:62<br>Pi-stacking: TRP:59;TRP:59
Rutin	Amylase	2qv4	-9.3	0.4	Hbond: GLN:63;GLN:63;ARG:195;ASP:197;ALA:198;LYS:200;GLU:233;ILE:235;HIS:299;HIS:305;HIS:305<br>Hydrophobic: TRP:58;TRP:58;TYR:62;TYR:151<br>Pi-stacking: TRP:59;TRP:59;TRP:59;HIS:201<br>Salt bridge: HIS:201;HIS:305
Syringic acid	Amylase	2qv4	-5.5	0.1	Hbond: GLN:63;ASP:300<br>Hydrophobic: TRP:59

4- hydroxy benzoic acid	BChE	6EQP	-5.9	1.1	Hbond: GLY:115;GLY:115<br>Pi-stacking: TRP:82;TRP:82
Caffeic acid	BChE	6EQP	-6.7	0.9	Hbond: ASP:70;SER:79;GLY:115;GLU:197;GLU:197<br>Hydrophobic: TRP:82;TRP:82<br>Pi-stacking: TRP:82
Chlorogenic acid	BChE	6EQP	-8.1	5.1	Hbond: ASN:83;GLY:115;GLY:115;THR:120;THR:120;TYR:128;GLU:197<br>Hydrophobic: TRP:82;THR:120<br>Pi-stacking: TRP:82
Hyperoside	BChE	6EQP	-10.5	1.1	Hbond: ASP:70;ASP:70;GLY:78;TRP:82;GLY:115;GLY:116;GLY:117;TYR:128;TYR:128;SER:198;LEU:286<br>Pi-stacking: PHE:329;PHE:329;TYR:332
Isoquercitrin	BChE	6EQP	-10.5	0.5	Hbond: ASP:70;GLY:78;SER:79;TRP:82;GLY:115;TYR:128;GLU:197;SER:198;SER:198;SER:287;HIS:438<br>Pi-stacking: PHE:329;PHE:329;TYR:332
Kaempferol-3-O-glucoside	BChE	6EQP	-10.4	0.1	Hbond: ASP:70;GLY:78;TRP:82;GLY:115;GLY:115;TYR:128;TYR:128;SER:198;SER:198<br>Pi-stacking: PHE:329;PHE:329;TYR:332
Quercetin	BChE	6EQP	-8.9	0.9	Hbond: GLY:116;GLY:117;ALA:199;LEU:286;SER:287<br>Hydrophobic: LEU:286;PHE:398<br>Pi-stacking: TRP:82;TRP:231;TRP:231;PHE:329;HIS:438
Rutin	BChE	6EQP	-9.4	1.1	Hbond: ASP:70;TRP:82;TRP:82;TRP:82;ASN:83;GLY:115;GLY:115;THR:120;GLU:197;PRO:285;TRP:430;HIS:438;TYR:440<br>Hydrophobic: ALA:328;PHE:329;PHE:329
Syringic acid	BChE	6EQP	-5.9	4.4	Hbond: HIS:438<br>Hydrophobic: TRP:82<br>Pi-stacking: TRP:82
4- hydroxy benzoic acid	Glucosidase	7KBJ	-6.0	1.1	Hbond: ARG:35;LYS:288;ASN:294;TRP:331<br>Hydrophobic: TRP:36;TRP:331
Caffeic acid	Glucosidase	7KBJ	-5.8	0.6	Hbond: TYR:246;GLU:248;TYR:291<br>Hydrophobic: LEU:252;LEU:252

Chlorogenic acid	Glucosidase	7KBJ	-8.4	0.4	Hbond: GLN:247;LYS:283;GLU:287;SER:290;ARG:334;ARG:352<br>Hydrophobic: LEU:252<br>Salt bridge: LYS:283
Hyperoside	Glucosidase	7KBJ	-7.0	7.0	Hbond: GLN:247;LYS:283;TYR:291;PHE:350;PHE:350;ARG:352;ARG:352;ARG:352<br>Hydrophobic: LEU:252;LEU:252;ILE:257;TYR:291<br>Salt bridge: ARG:352<br>Pi-cation: ARG:352
Isoquercitrin	Glucosidase	7KBJ	-8.3	3.8	Hbond: ASP:251;LEU:252;GLY:253;GLY:254;MET:289;TYR:291;ARG:334;ARG:334;ARG:352;ARG:352<br>Hydrophobic: TRP:331<br>Pi-stacking: TYR:291<br>Salt bridge: ARG:352
Kaempferol-3-O-glucoside	Glucosidase	7KBJ	-8.3	0.6	Hbond: ILE:250;LEU:252;GLY:253;GLY:254;ASN:265;MET:289;TYR:291;ARG:334;ARG:334;ARG:352<br>Hydrophobic: TRP:331<br>Pi-stacking: TYR:291<br>Salt bridge: ARG:352
Quercetin	Glucosidase	7KBJ	-8.4	1.1	Hbond: GLY:253;ASN:265;MET:289;TYR:291;TYR:291;ARG:334;ARG:352<br>Hydrophobic: ILE:257;TYR:291
Rutin	Glucosidase	7KBJ	-8.8	6.0	Hbond: GLN:247;GLU:248;GLU:248;GLY:264;TYR:291;TYR:291;ARG:334;TYR:348;PHE:350;ARG:352<br>Hydrophobic: PHE:237;LEU:252;LEU:344;PHE:350;ARG:352<br>Salt bridge: LYS:283;ARG:352;ARG:352<br>Pi-cation: LYS:283;ARG:352
Syringic acid	Glucosidase	7KBJ	-5.8	0.8	Hbond: ASP:251;LEU:252;GLY:253;GLY:254;GLY:266;GLU:287;GLU:287;SER:290<br>Hydrophobic: LEU:252<br>Salt bridge: LYS:283;ARG:352
4- hydroxy benzoic acid	IL6	5FUC	-5.0	0.4	Hydrophobic: LYS:66;MET:67;GLU:172
Caffeic acid	IL6	5FUC	-5.2	0.7	Hbond: CYS:73;GLN:75;GLN:183<br>Hydrophobic: PHE:78;ARG:179;GLN:183<br>Salt bridge: ARG:179
Chlorogenic acid	IL6	5FUC	-6.4	1.5	Hbond: GLN:75;GLN:75;ARG:179;GLN:183<br>Hydrophobic: GLN:75;GLN:75;GLN:183

Hyperoside	IL6	5FUC	-7.0	0.5	Hbond: PRO:65;GLU:172;GLU:172;GLN:175;SER:176;ARG:179;ARG:179<br>Hydrophobic: MET:67;PHE:74;GLU:172;PHE:173
Isoquercitrin	IL6	5FUC	-7.1	0.9	Hbond: SER:169;GLU:172;GLN:175;SER:176;SER:176;ARG:179;ARG:179<br>Hydrophobic: MET:67;GLU:172;PHE:173
Kaempferol-3-O-glucoside	IL6	5FUC	-7.0	0.2	Hbond: PRO:65;GLN:175;SER:176;SER:176;ARG:179;ARG:179<br>Hydrophobic: LYS:66;MET:67;PHE:173
Quercetin	IL6	5FUC	-6.6	1.0	Hbond: ARG:179;ARG:179;GLN:183<br>Hydrophobic: PHE:74;GLN:75;ARG:179<br>Pi-stacking: PHE:74;PHE:74
Rutin	IL6	5FUC	-7.3	1.1	Hbond: PRO:65;MET:67;GLN:75;SER:169;GLU:172;SER:176<br>Hydrophobic: PHE:74;GLN:75
Syringic acid	IL6	5FUC	-4.8	5.3	Hbond: GLN:75;GLN:183<br>Hydrophobic: ARG:179<br>Salt bridge: ARG:182
4- hydroxy benzoic acid	ILR6	5fuc	-5.6	1.0	Hbond: GLN:281<br>Hydrophobic: PRO:107;ALA:192;LEU:195;TYR:230;GLU:277<br>Salt bridge: ARG:104
Caffeic acid	ILR6	5fuc	-5.9	0.5	Hbond: ARG:104;ARG:104;VAL:161;PRO:162;GLY:164;ASP:165;GLN:281<br>Hydrophobic: PRO:107;ALA:192;TYR:230
Chlorogenic acid	ILR6	5fuc	-7.2	0.4	Hbond: PRO:107;PRO:162;PHE:229;GLU:277;GLU:278;GLN:281<br>Hydrophobic: PHE:229;TYR:230;PHE:279<br>Salt bridge: ARG:104
Hyperoside	ILR6	5fuc	-7.5	0.9	Hbond:<br>ARG:104;ARG:104;PRO:107;PRO:162;GLU:163;GLY:164;ASP:165;GLN:190;GLN:190;GLN:281<br>Pi-stacking: TYR:230
Isoquercitrin	ILR6	5fuc	-7.5	0.9	Hbond: ARG:104;GLY:164;GLY:164;GLN:190;ALA:192;TYR:230;GLU:277;GLU:277;GLN:281
Kaempferol-3-O-glucoside	ILR6	5fuc	-7.4	0.3	Hbond: ARG:104;PRO:162;GLY:164;GLY:191;GLU:277<br>Hydrophobic: ALA:192
Quercetin	ILR6	5fuc	-7.4	1.0	Hbond: ARG:104;ARG:104;GLY:164;ASP:165;ASP:165;ALA:192;GLN:281<br>Hydrophobic: PRO:107;PHE:279

Rutin	ILR6	5fuc	-8.0	0.9	Hbond: ARG:104;ARG:104;PRO:107;PRO:162;GLY:164;GLU:277;GLU:277;GLN:281;GLN:281<br>Hydrophobic: PRO:107;GLU:163;ALA:192;LEU:195;PHE:229;TYR:230
Syringic acid	ILR6	5fuc	-5.4	1.1	Hbond: GLY:164;GLY:191;GLU:277;GLU:277<br>Hydrophobic: ALA:192
4- hydroxy benzoic acid	PTGS2	5F19	-6.4	0.8	Hbond: THR:206;TYR:385;HIS:388<br>Hydrophobic: ALA:202;GLN:203;GLN:203;TYR:385;TRP:387;TRP:387;LEU:390
Caffeic acid	PTGS2	5F19	-7.2	13.2	Hbond: GLN:203;TYR:385<br>Hydrophobic: GLN:203;GLN:203<br>Salt bridge: HIS:207;HIS:386
Chlorogenic acid	PTGS2	5F19	-8.9	1.1	Hbond: ARG:44;GLY:45;GLY:45;GLY:135;GLU:465<br>Hydrophobic: PRO:153;PRO:156<br>Salt bridge: HIS:39;LYS:468
Hyperoside	PTGS2	5F19	-8.9	0.9	Hbond: ASN:34;ASN:34;HIS:39;CYS:47;SER:49;LYS:137<br>Hydrophobic: PRO:153;PRO:154
Isoquercitrin	PTGS2	5F19	-8.9	0.7	Hbond: GLN:203;PHE:210;THR:212;THR:212;TYR:385;HIS:388;HIS:388;HIS:388<br>Hydrophobic: VAL:447;VAL:447<br>Pi-stacking: HIS:207;HIS:214<br>Salt bridge: HIS:207;HIS:386;HIS:388<br>Pi-cation: HIS:207;HIS:388
Kaempferol-3-O-glucoside	PTGS2	5F19	-8.5	0.9	Hbond: ASN:34;SER:49;SER:49;SER:49;GLY:135;LYS:137<br>Hydrophobic: PRO:153;PRO:154;PRO:156
Quercetin	PTGS2	5F19	-9.5	4.7	Hbond: CYS:47;GLY:135;GLU:465;GLU:465<br>Hydrophobic: LEU:152;PRO:153
Rutin	PTGS2	5F19	-9.9	3.9	Hbond: ALA:199;ALA:199;GLN:203;HIS:214;ASN:382;HIS:386;TRP:387;LYS:446;VAL:447;GLN:454<br>Hydrophobic: VAL:291;TYR:385;TRP:387;ALA:450<br>Pi-stacking: HIS:207;HIS:214<br>Salt bridge: HIS:207;HIS:207;HIS:386;HIS:388
Syringic acid	PTGS2	5F19	-6.8	26.8	Hbond: THR:206;HIS:207;TYR:385;HIS:388<br>Hydrophobic: GLN:203

4- hydroxy benzoic acid	Tyrosinase	6QXD	-5.6	0.7	Hbond: GLU:195;MET:215<br>Hydrophobic: VAL:218<br>Pi-stacking: HIS:208<br>Salt bridge: HIS:204
Caffeic acid	Tyrosinase	6QXD	-6.0	0.8	Hbond: HIS:42;GLY:216<br>Hydrophobic: VAL:218<br>Pi-stacking: HIS:208<br>Salt bridge: ARG:209
Chlorogenic acid	Tyrosinase	6QXD	-6.6	1.0	Hbond: HIS:60;GLY:216;VAL:217<br>Hydrophobic: HIS:208;VAL:218<br>Pi-stacking: HIS:208<br>Salt bridge: ARG:209;ARG:209
Hyperoside	Tyrosinase	6QXD	-5.0	5.6	Hbond: ASN:205;ARG:209;ARG:209;ARG:209;GLY:216<br>Hydrophobic: VAL:217;VAL:218
Isoquercitrin	Tyrosinase	6QXD	-5.4	1.1	Hbond: ASN:205;ASN:205;ARG:209;ARG:209;ARG:209<br>Hydrophobic: VAL:217;VAL:218
Kaempferol-3-O-glucoside	Tyrosinase	6QXD	-5.1	0.8	Hbond: ASN:205;HIS:208;ARG:209<br>Hydrophobic: VAL:217;VAL:218
Quercetin	Tyrosinase	6QXD	-6.1	3.4	Hbond: ARG:209;VAL:217<br>Pi-stacking: HIS:208<br>Pi-cation: ARG:209
Rutin	Tyrosinase	6QXD	-4.7	3.9	Hbond: HIS:60;ASN:205<br>Hydrophobic: HIS:60;VAL:218;ALA:221<br>Pi-stacking: HIS:208<br>Salt bridge: HIS:208;ARG:209
Syringic acid	Tyrosinase	6QXD	-6.2	0.8	Hbond: GLU:195;ASN:205;GLY:216<br>Hydrophobic: VAL:218<br>Pi-stacking: HIS:208<br>Salt bridge: HIS:60;HIS:204

References

Grochowski, D.M., Uysal, S., Aktumsek, A., Granica, S., Zengin, G., Ceylan, R., Locatelli, M., Tomczyk, M., 2017. In vitro enzyme inhibitory properties, antioxidant activities, and phytochemical profile of *Potentilla thuringiaca*. Phytochemistry letters 20, 365-372.

Livak, K.J., Schmittgen, T.D., 2001. Analysis of relative gene expression data using real-time quantitative PCR and the 2− ΔΔCT method. methods 25(4), 402-408.

Orlando, G., Chiavaroli, A., Adorisio, S., Delfino, D. V., Brunetti, L., Recinella, L., Leone, S., Zengin, G., Acquaviva, A., Angelini, P., Flores, G. A., Venanzoni, R., Di Simone, S. C., Di Corpo, F., Mocan, A., Menghini, L., & Ferrante, C. (2021). Unravelling the Phytochemical Composition and the Pharmacological Properties of an Optimized Extract from the Fruit from *Prunus mahaleb* L.: From Traditional Liqueur Market to the Pharmacy Shelf. *Molecules (Basel, Switzerland)*, *26*(15), 4422. https://doi.org/10.3390/molecules26154422

Mustafa, A.M., Angeloni, S., Abouelenein, D., Acquaticci, L., Xiao, J., Sagratini, G., Maggi, F., Vittori, S., Caprioli, G., 2022. A new HPLC-MS/MS method for the simultaneous determination of 36 polyphenols in blueberry, strawberry and their commercial products and determination of antioxidant activity. Food Chemistry 367, 130743.

Uysal, S., Zengin, G., Locatelli, M., Bahadori, M. B., Mocan, A., Bellagamba, G., . . . Aktumsek, A. (2017). Cytotoxic and enzyme inhibitory potential of two Potentilla species (P. speciosa L. and P. reptans Willd.) and their chemical composition. *Frontiers in pharmacology, 8*, 290.
